# Limitations of non-polarizable force fields in describing anion binding poses in non-polar synthetic hosts†

**DOI:** 10.1039/d3cp00479a

**Published:** 2023-06-20

**Authors:** David Seiferth, Stephen J. Tucker, Philip C. Biggin

**Affiliations:** a Clarendon Laboratory, Department of Physics, University of Oxford Oxford OX1 3PU UK; b Structural Bioinformatics and Computational Biochemistry, Department of Biochemistry, University of Oxford Oxford OX1 3QU UK philip.biggin@bioch.ox.ac.uk; c Kavli Institute for Nanoscience Discovery, University of Oxford Oxford UK

## Abstract

Transmembrane anion transport by synthetic ionophores has received increasing interest not only because of its relevance for understanding endogenous anion transport, but also because of potential implications for therapeutic routes in disease states where chloride transport is impaired. Computational studies can shed light on the binding recognition process and can deepen our mechanistic understanding of them. However, the ability of molecular mechanics methods to properly capture solvation and binding properties of anions is known to be challenging. Consequently, polarizable models have been suggested to improve the accuracy of such calculations. In this study, we calculate binding free energies for different anions to the synthetic ionophore, biotin[6]uril hexamethyl ester in acetonitrile and to biotin[6]uril hexaacid in water by employing non-polarizable and polarizable force fields. Anion binding shows strong solvent dependency consistent with experimental studies. In water, the binding strengths are iodide > bromide > chloride, and reversed in acetonitrile. These trends are well captured by both classes of force fields. However, the free energy profiles obtained from potential of mean force calculations and preferred binding positions of anions depend on the treatment of electrostatics. Results from simulations using the AMOEBA force-field, which recapitulate the observed binding positions, suggest strong effects from multipoles dominate with a smaller contribution from polarization. The oxidation status of the macrocycle was also found to influence anion recognition in water. Overall, these results have implications for the understanding of anion host interactions not just in synthetic ionophores, but also in narrow cavities of biological ion channels.

## Abstract 2

We investigate how using polarizable or non-polarizable force fields influences the energetics and binding mode for different anions with synthetic ionophores. Our results have important implications for studying anions in biological channels.

## Graphical abstract

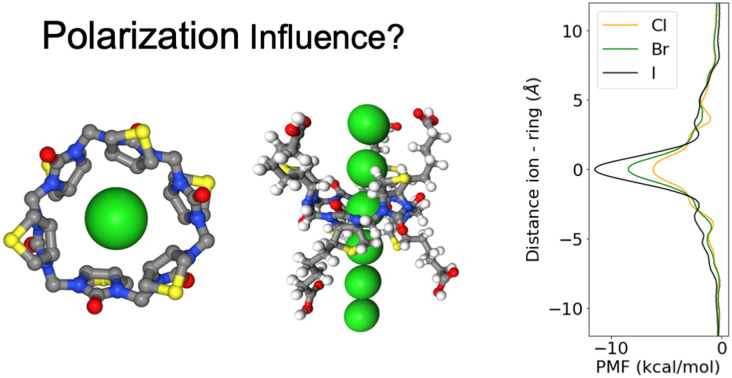

## 1. Introduction

Biological ion channels and transporters are integral to the healthy function of all living cells. They allow the selective permeation of ions and water across cell membranes to control cellular electrical activity and hence maintain cellular and ionic homeostasis. Chloride is the most abundant anion in living organisms and is involved in processes such as controlling the membrane potential, the cell volume and pH balance.^1^ Dysregulation of chloride ions can also result in numerous disease states, the most common human disorder being cystic fibrosis.^2^ Transmembrane anion transport by synthetic ionophores therefore has therapeutic potential to supplement defective ion transport^3^ and there are also artificial chloride channels with reported anticancer activity.^4^ Ion transport is usually highly selective for specific ions and is essential to maintain ion concentration gradients across biological membranes. The interaction of both biological and synthetic carriers with both anions and cations has been investigated for a long time, especially now that so many structures are available, but the majority of studies have focused on interactions with cations. The interaction of carriers, synthetic or otherwise, with anions is more challenging because it must solve the problem of the relatively high solvation energy of anions compared to similar size cations. Furthermore, the presence of water can negate the stabilizing intermolecular interactions that enable anion binding.^5^

**Fig. 1:**
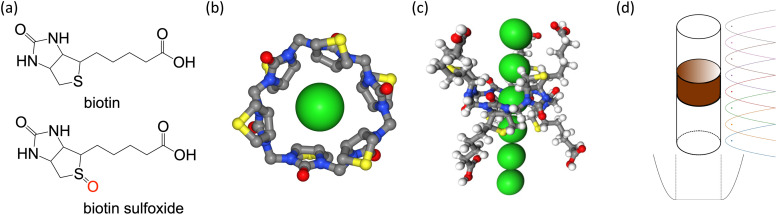
(a) Biotin and biotin sulfoxide unit. (b) Chloride (green sphere) inside the cavity of biotin[6]uril macrocycle. Side chains and hydrogen atoms are not shown. The centre of the cavity is defined as the centre of geometry of the 12 inside-pointing carbon atoms (grey spheres) from the convex side of the biotin units. (c) Visualisation of the reaction coordinate for PMF calculations. The distances of neighbouring chloride ions to the centre of the cavity differ by 4 Å as illustrated by the green spheres along the reaction coordinate. (d) System set-up. The position and orientation of the ion carrier (brown cylinder) is preserved by restraining three carbon atoms. Umbrella potentials are applied along the *z*-axis to ensure sufficient sampling along the reaction coordinate (distance of the ion to the cavity centre). A flat-bottom potential in the *xy*-plane prevents the ion from moving far away from the carrier.

In a recent study, Lisbjerg *et al.* presented a macrocyclic anion receptor called biotin[6]uril.^6–8^ In this carrier, six biotin units form a ring *via* their nitrogen atoms. Twelve CH-groups from the convex side of each biotin unit point into the centre of the ring and form a cavity as depicted in Fig. 1. The CH-bonds are slightly polarised due to inductive electron withdrawal of the urea moieties and make the cavity slightly hydrophobic.^6^ The biotin monomer shows no recognition of anions.^8^ It was found that the carrier with methylated side chains preferentially binds smaller halides such as chloride and bromide over the larger halide iodide in the nonpolar solvent acetonitrile.^6^ In water, the trend is reversed and the biotin macrocycle has a higher binding affinity for larger anions such as iodide and bromide over chloride. Solvent effects also play an important role in anion selectivity of receptor molecules. Water is a strongly competitive solvent and can negate most of the stabilizing intermolecular forces that enable the binding of anions in host-guest systems.^5^ Furthermore, the sulfide moiety of each biotin monomer of the macrocycle can be oxidized (Fig. 1a). The resulting sulfoxide moiety becomes more electron withdrawing than sulfide and changes the cavity potential of the macrocycle.^8^ These properties therefore make this carrier an interesting case study for different computational approaches that appear to influence anion interactions.

Anions can be ordered according to their capability to stabilize proteins from water in the so-called Hofmeister series: F < Cl < Br< I.^9^ Smaller, hard halides, namely fluoride and chloride are less polarizable and have a stronger and more well-defined solvation shell. They decrease the solubility of nonpolar molecules (‘salting out’) and are less prone to migration towards hydrophobic interfaces. However, the larger, softer and more polarizable anions such as bromide and iodide have a weaker and less well-defined solvation shell. They increase the solubility of nonpolar molecules (‘salting in’) and are more prone to migration towards hydrophobic interfaces.

In this work, we have investigated electrostatic effects, including induced polarization, by calculating the free energy of binding of different anions to biotin macrocycles solvated in water and in acetonitrile using molecular dynamics (MD) simulation techniques. In principle, the free energy can be obtained directly by counting bound and unbound configurations in long unbiased simulations. In practice, this is rarely feasible due to energy barriers between phase space regions that prevent exhaustive sampling of all relevant phase space configurations in finite time. This is generally referred to as the problem of quasi-nonergodicity.^10^*In silico* calculations of free energy rely on three components: a model Hamiltonian, a sampling protocol, and a method to estimate the free energy difference.^10^ A model Hamiltonian is used to compute energies and forces and to ensure that all configurations are sampled with the correct relative probability. Here, we use two different types of classical Hamiltonians, generally referred to as force fields. They mainly differ in the way they describe non-bonded interactions; namely van der Waals and electrostatic interactions.

We first ascertain what level of error can be expected in both fixed charged and polarizable models. The ability of molecular mechanics methods to properly capture solvation and binding properties of ions is known to be problematic: explaining the high selectivity for fluoride over chloride despite their identical charge and the size similarity in fluoride-selective ion channels^11^ or the potassium/sodium selectivity of potassium channels^12^ is challenging. Ion parameters are usually calibrated against reference data on ion–water interactions, but the transferability of these parameters to interactions of ions with other functional groups is limited.^13^

There are different methods for improving the accuracy of anion MD properties by changing non-bonded interactions in a molecular mechanics force field. Li *et al.* re-optimized the Lennard-Jones parameters for van der Waals interactions to reproduce hydration free energies within the AMBER force field.^14^ Parameters in the non-polarizable CHARMM force field systematically underestimate the interaction of chloride with proteins and lipids.^15^ Hence, Orabi *et al.* formulated a series of pair-specific Lennard-Jones parameters that were systematically calibrated against available experimental data as well as *ab initio* geometry optimizations and energy evaluations. Instead of tuning the van der Waals interactions, one can also change electrostatic interactions: Standard additive force fields can be modified to include polarization in a mean-field fashion. The electronic continuum correction (ECC) method models the polarizability of ions in an averaged static way by scaling the ionic charges by a factor of 
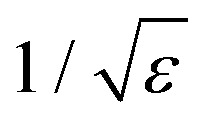
, where *ε* is the dielectric constant of the solvent^16^ and has been used to examine halide-nanotube interactions recently.^17^ The ECC method cannot however model how the strength of dipoles changes in response to their local environment over time.^18^

Conventional pairwise additive force fields use the two-body Coulomb potential to describe charge–charge interactions and cannot take into account the effect of induced polarization. A polarizable atomic multipole model such as AMOEBA describes the charge distribution of atoms not only with partial charges but also includes higher multipole moments and can model the effect of induced polarization.

The Drude oscillator model implemented in the CHARMM Drude force field is another approach that incorporates polarization effects into classical MD simulations. Indeed, Drude oscillators have previously been used to investigate the effects of polarization on the hydration of chloride.^19^

The second component of free energy calculations is the sampling protocol. We are interested in the (Gibbs) free energy difference between two states A and B that represent the bound and the unbound state of an ion with an ion carrier in an isothermal–isobaric (*NPT*) ensemble.

The free energy difference:1
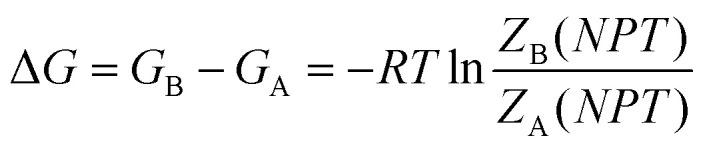
depends on the ratio of the partition functions *Z*_*i*_(*NPT*) of the systems A and B.

Here, we use a physical pathway method to measure the reversible work (the potential of mean force (PMF)) to transfer the ion from the bulk into the cavity of the host molecule. The relationship between the standard free energy of binding and the change in the PMF on binding is often assumed to be equivalent.^20^ Here, we calculate the binding free energy as the difference between the bulk and the minimum at the binding site as computed by the PMF. We find that in hydrophobic and narrow cavities, the binding free energies and binding poses are highly sensitive to whether the force field can capture multipole and polarization effects.

## 2. Methods

### Ion parameters

Within molecular mechanics force fields, the 12-6 Lennard-Jones potential is the most commonly used potential to model van der Waals interactions. In simulations with Parsley,^21^ which we selected as the non-polarizable force field, we used the ion parameters from Li *et al.*, which were parameterized to reproduce hydration free energies.^14^ Fig. S1 (ESI†) shows the 12-6 Lennard-Jones potential for the four halide anions. We also explored the Zhang *et al.*^22^ parameter set. However, it is worth noting these parameters were recently criticized with respect to the dangers of over-fitting the van der Waals radii.^23^ The chloride parameters from Li *et al.*,^14^ 2015 relate both the radius and the well depth *via* a so-called “noble-gas” curve, which is a fit based on the vdW parameters of noble gas atoms to correlate the radius and the well depth in the LJ equation.

Many non-polarizable force fields are fitted to reproduce gas-phase conformational energies and geometries. Non-bonded interactions are particularly important in condensed phase simulations. Of the non-bonded interactions, electrostatics models are often polarized beyond what would be expected in the gas phase.^24^ The transferability of parameters for pairwise additive force fields can be limited due to their fixed charges and their inability to model the effect of induced polarization. The Parsley force field uses a fixed partial charge model and a Coulomb potential to describe electrostatic interactions. A fixed partial charge model corresponds to a multipole expansion where dipole, quadrupole and higher-order terms are truncated.

Compared to additive force fields, the AMOEBA force field^25,26^ uses different potentials for non-bonded interactions. van der Waals interactions are described by a buffered 14-7 potential. The AMOEBA force field takes multipoles up to quadrupoles at each atomic centre into account and truncates octupole and higher moments. The electrostatic energy in AMOEBA includes contributions from both permanent and induced multipoles. Only the dipole moment is treated as inducible. Monopole (*i.e.* charge) and quadrupole moments are invariable. The inclusion of explicit dipole polarization allows the AMOEBA model to respond to changing or heterogeneous molecular environments.^25^ An external field distorts the atomic electron density. A classical point dipole moment is induced at each polarizable atomic site according to the electric field felt by that site in AMOEBA. In order to prevent the divergence of induced dipole moments at short interatomic distances, the so-called polarization catastrophe, the AMOEBA force field incorporates Thole's damped interaction method.^27^ Polarization interaction at very short range is damped by smearing out the atomic multipole moments. We followed the POLTYPE protocol^28^ for the parameterization with the AMOEBA force field. The parameterization process is described in more detail in the ESI,† Text S1.

### Simulation protocol

Simulations with the AMOEBA force field were carried out with Tinker9.^29^ We used similar parameters as in a recent study using AMOEBA for calculating PMFs of an ion channel;^30^ Time integration was performed using the reversible reference system propagator algorithm (RESPA)^31^ with an outer time step of 2 fs and an inner time step of 0.25 fs. The van der Waals and electrostatic multipole forces were updated in intervals of the outer time step and all other forces were evaluated at each inner time step. The self-consistent induced dipole moments between iterations were converged to *ε* < 0.000001 D. The Ewald cut-off was set to 7.0 Å and the van der Waals cut-off to 10 Å. The temperature of the simulation system was maintained at a temperature of 298.15 K using the Bussi thermostat^32^ with 0.1 ps coupling time. A Monte Carlo barostat was used to maintain a pressure of 1 atm with 2 ps coupling time. For polarizable atomic multipole interactions, Tinker provides regular Ewald summation method to include long range electrostatic interactions in a system with periodic boundary conditions.

For Parsley simulations, GROMACS^33^ was used as the simulation engine using the leap-frog algorithm with a 2 fs time step. Electrostatics were computed with the particle mesh Ewald method^34,35^ with a 14 Å cut-off. The velocity rescaling thermostat^32^ was used with a 0.1 ps coupling time. The pressure was maintained by the Parrinello–Rahman barostat^36^ with 2 ps coupling time for production simulations. The isothermal compressibility for water was set to 4.5 × 10^−5^ bar^−1^ and for acetonitrile was set to 10.8 × 10^−5^ bar^−1^.^37^

### Potential of mean force calculations

The potential of mean force (PMF) was calculated with umbrella sampling for the two different force fields (Parsley, AMOEBA), two different solvents (water and acetonitrile) and three halide anions (chloride, bromide, iodide). The distance between the *z*-coordinates of the centre of mass (COM) of the cavity (defined by the twelve carbon atoms) and the *z*-coordinate of the ion was set as the reaction coordinate as depicted in Fig. 1(c). Position restraints to three carbon atoms of the cavity were applied to retain the orientation of the carrier during the simulations while still allowing the cavity full flexibility. The *z*-coordinate of the ion was harmonically restrained to a specific distance with respect to the COM of the cavity in each umbrella window to ensure sampling along the reaction coordinate, see Fig. 1(d). For Parsley simulations, we simulated 25 × 25 ns windows with 1 Å spacing for the distance bias of 500 kJ mol^−1^ nm^−2^. We sampled the reaction coordinate between 0 Å < *z* < 25 Å. For some systems, additional windows with higher bias were needed to ensure sufficient sampling in energy barrier regions along the reaction coordinate. In addition to the distance restraint of the *z*-coordinate, we introduced flat bottom restraints in the *xy*-plane to force the ion to stay within a cylinder of radius *R*_cyl_ = 2.5 Å or 3.5 Å. Within the cylinder, the ion can move freely and explore the phase space. Outside of the cylinder, there are high harmonic restraints of 5000 kJ mol^−1^ nm^−2^, forcing the ion back to the cylinder. This cylinder ensures that the ion cannot move laterally away from the carrier when its *z*-coordinate is restrained to a value which corresponds to a region where binding and unbinding events are very frequent. Unbinding events in the *xy*-plane are irreversible on the time scale of the window (25 ns) and affect convergence of the potential of mean force calculations. In long unbiased simulations (500 ns), one can observe a handful of binding and unbinding events. Simulating each window for 500 ns is not feasible and hence we study only the binding and unbinding dynamics along the *z*-coordinate and restrain the movement of the ion in the *xy*-plane to an area of the size of the cavity. This allows us to calculate the area sampled by the ion orthogonal to the reaction coordinate and limits the sampling required in each window.^20^

The binding free energy

2 Δ*G* = −*RT *ln(*C*^0^*K*_b_)

with equilibrium binding constant *K*_b_ and standard concentration *C*^0^ = 1/1661 Å^−3^ can be corrected by the term

3 Δ*G*_corr_ = −*RT *ln(π*R*_cyl_^2^/*A*^0^)

where π*R*_cyl_^2^ is the surface of the cylinder.^20,38^ When the surface of the cylinder equals the so-called standard surface *A*^0^, the correction is zero. We define the standard surface as *A*^0^ = π*r*_0_^2^ = 170 Å^2^ if *r*_0_ denotes the radius of a sphere with standard volume 1661 Å^3^.

The first nanosecond of each window was discarded as equilibration time. The remaining 24 ns were divided into three sub-windows of 8 ns length defining three data sets. From each of the three sets the PMF was calculated. The bulk region was defined as the zero point. The minimum of the PMF averaged over three data sets is interpreted as binding free energy and the standard deviation of the minimum as the error. For AMOEBA simulations, the set-up is similar except for that we bias the *z*-coordinate of the ion with 2 kcal mol^−1^ Å^−2^ and 0.5 Å spacing in 60 windows between −15 Å < *z* < 15 Å distance to the COM of the cavity. Each window was simulated for 13 ns of which the first nanosecond was discarded as equilibration time. The remaining simulation time was divided into three parts to define three data sets. The histogram overlap for each PMF was also checked to ensure good overlap between windows. Example overlap data for this is shown in the ESI† (Fig. S2) for the first set of PMFs calculated.

## 3. Results and discussion

### Ion interactions with methylated biotin[6]uril in acetonitrile

**Fig. 2:**
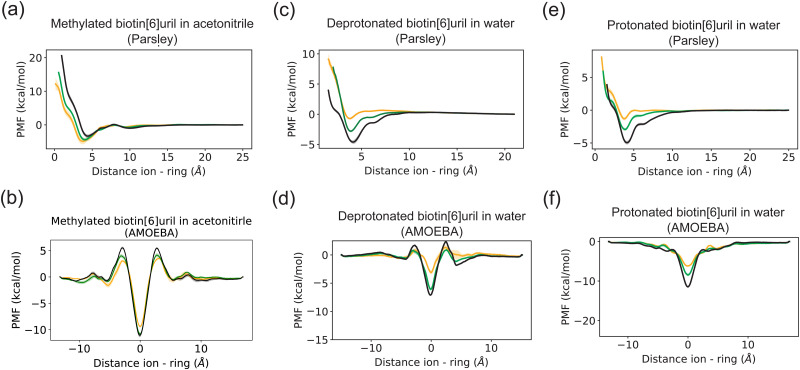
Potential of mean force (PMF) of ions and a biotin macrocycle with methylated side chains in (a and b) acetonitrile and an unmethylated macrocycle in water in deprotonated (c and d) and protonated (e and f) forms for two different force fields. Each PMF here corresponds to the average of three data sets. Error bars represent one standard deviation. Two representative binding poses are depicted in Fig. 3. The histogram overlap for these PMFs can be found in the Fig. S2 (ESI†). Chloride is depicted in yellow, bromide in green and iodide in black.

To calculate the free energy of binding between different ions and hosts, we employed molecular dynamics simulations with a non-polarisable additive force field (Parsley) and the polarizable AMOEBA force field. As calculations with GAFF revealed similar PMF profiles (Fig. S3, ESI†) we keep the focus here on the difference between Parsley (as representative of a non-polarizable force field) with AMOEBA. We estimated the PMF of the interaction between the biotin macrocycle and different anions in acetonitrile and water in Fig. 2. The estimated values for the binding free energies are summarized in Tables 1 and 2 and are compared to experimental free energy values obtained using with isothermal titration calorimetry (ITC).^6,8^ The solvent affects the binding affinities significantly.

**Table 1:** Estimates for the binding free energies for biotin[6]uril hexamethyl ester and different anions in acetonitrile. Binding free energies are estimated by the difference between minimum and bulk PMF values. The corresponding PMFs are depicted in Fig. 2

	Chloride	Bromide	Iodide
ITC in acetonitrile^a^	−6.3	−6.3	−4.2
PMF (Parsley)	−4.0 ± 0.9	−3.5 ± 0.5	−2.4 ± 0.5
PMF (AMOEBA)	−8.1 ± 0.2	−9.5 ± 0.2	−9.8 ± 0.4

a Note Lisberg *et al.* do not report error estimates.

**Table 2:** Estimates for the binding free energies in kcal mol^−1^ for biotin[6]uril hexaacid ester and different anions in water. Binding free energies are estimated by the difference between minimum and bulk PMF. The corresponding PMFs are depicted in Fig. 2. In water, the acid could exist as protonated or deprotonated and thus both forms were considered. The PMF minima are corrected by eqn (3) to take the orthogonal degrees of freedom into account (note that the binding energy is positive for chloride and the deprotonated host when using the Parsley force field)

	Chloride	Bromide	Iodide
ITC in water	−1.5	−3.8	−4.8
Deprotonated PMF (Parsley)	+0.6 ± 0.2	−1.5 ± 0.2	−3.3 ± 0.3
Deprotonated PMF (Amoeba)	−1.8 ± 0.3	−4.8 ± 0.2	−5.8 ± 0.5
Protonated PMF (Parsley)	−0.4 ± 0.3	−2.0 ± 0.2	−4.1 ± 0.3
Protonated PMF (Amoeba)	−4.9 ± 0.2	−7.1 ± 0.4	−10.2 ± 0.2

According to the ITC data from Lisbjerg *et al.*,^6^ when the macrocycle is in the nonpolar solvent acetonitrile, chloride and bromide have a higher binding affinity than iodide, and in simulations with the non-polarizable Parsley force field we also observe a similar trend, *i.e.* smaller and harder anions bind better than larger softer anions as shown in Table 1. The binding affinities estimated with the polarizable AMOEBA force field are much greater, but no differences in anion selectivity are observed with all three halides appearing stably bound.

**Fig. 3:**
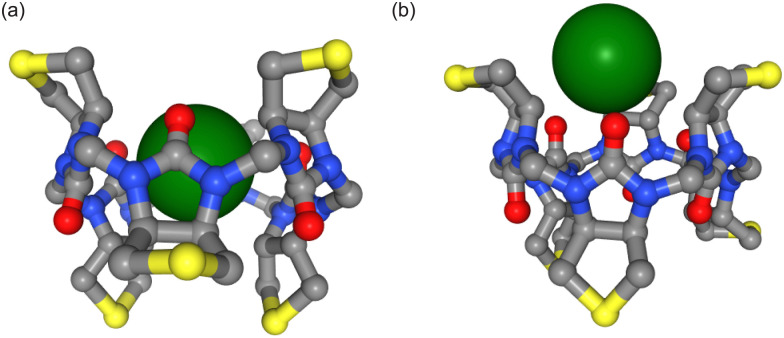
Different binding poses of ions (white spheres) within the biotin macrocycle. Side chains and hydrogen atoms are omitted for clarity. The distance between the centre of mass of the cavity (defined by 12 carbon atoms) and the ion is zero and 3.5 Å respectively in (a and b). Simulations with the AMOEBA force field predict binding within the cavity (a) whereas nonpolarizable simulations predict the favourable binding site to be slightly outside the cavity (b).

The inclusion of multipoles and polarization therefore does not appear to improve the correlation with the experimental ITC data. However, a closer examination reveals that the PMFs from Parsley and AMOEBA differ considerably (Fig. 2). In contradiction with the experimentally derived structure,^7^ Parsley suggests that ion binding within the cavity is not favorable. An ion within the cavity corresponds to a zero distance in the reaction coordinates shown in Fig. 2. However, for Parsley, the minima of the PMFs appear between 3–4 Å on the reaction coordinate (Fig. 2a, c, e and Fig. 3b). In these non-polarizable simulations, an ion moving from the PMF minimum closer to the cavity must overcome a steep energy barrier. In marked contrast, the energetic landscape along the reaction coordinate in simulations with the AMOEBA force field demonstrate that ions within the cavity are in a favorable position and the origin of the reaction coordinate corresponds to the energetic minimum (Fig. 2b, d, f and 3a). Ions coming from the bulk must overcome an energetic barrier of only ∼3 kcal mol^−1^ to bind symmetrically in the cavity.

### Ion interactions with unmethylated biotin[6]uril in water

The trends in selectivity observed in acetonitrile are reversed in water. According to ITC experiments, the larger and softer iodide binds better to the unmethylated biotin macrocycle in water than the smaller and harder bromide,^8^ and our simulations reproduce this trend. Larger, softer and more polarisable anions such as bromide and iodide have a weaker and less well-defined solvation shell than the smaller chloride (see anion hydration free energies – Table S1, ESI†). The hydration free energies of halide anions have a smaller magnitude in acetonitrile than in water, but the overall trend is the same in both solvents. The energetic landscape represented by the PMFs depicted in Fig. 2 has deeper wells for iodide than for bromide or chloride across all three force fields. Table 2 summarizes the binding free energies of ions with the carrier in water: The larger (and more polarizable) the ion, the higher the binding affinity thus reproducing a preferential binding for larger anions. Both force fields capture the overall trend, but once again, the AMOEBA force field predicts larger binding free energies than the non-polarizable Parsley.

### Ion interactions with unmethylated biotin-sulfoxide macrocycle in water

**Fig. 4:**
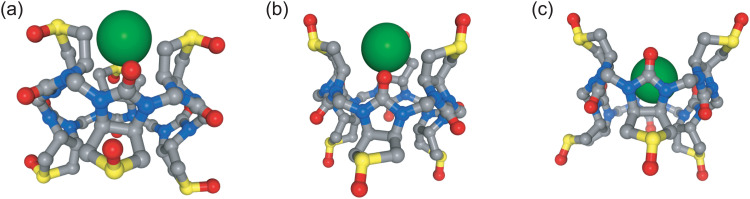
Non-central binding pose of chloride (green sphere) within the biotin-d-sulfoxide (a) and biotin-l-sulfoxide (b). Central binding pose of iodide within the biotin-l-sulfoxide macrocycle (c). Side chains and hydrogen atoms are omitted for clarity. The distance between the centre of mass of the cavity (defined by 12 carbon atoms) and the ion is 4 Å and corresponds to a free energy minimum.

The sulfide moiety of each biotin monomer of the macrocycle can be oxidized. There are two possible conformations as a result: the oxygen can point away from the cavity (d-isomer) or perpendicular to the cavity (l-isomer) or as depicted in Fig. 4.

**Fig. 5:**
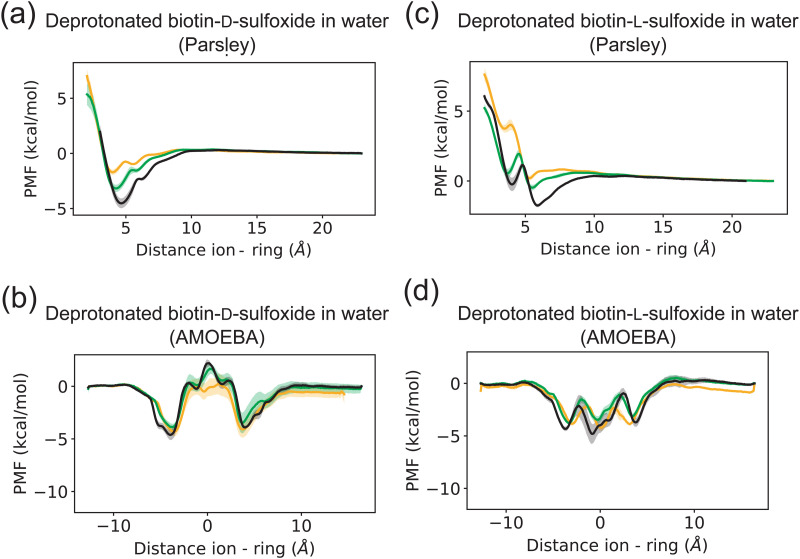
Potential of mean force (PMF) of ions and a deprotonated biotin sulfoxide macrocycle in two different conformations (d-conformer in (a) and (b), l-conformer in (c and d)) with unmethylated side chains in water for two different force fields (Parsley in the first row, AMOEBA in the second row). Each PMF here corresponds to the average of three data sets. The standard deviation of the three data sets is plotted as error bar. Chloride is depicted in yellow, bromide in green and iodide in black.

**Fig. 6:**
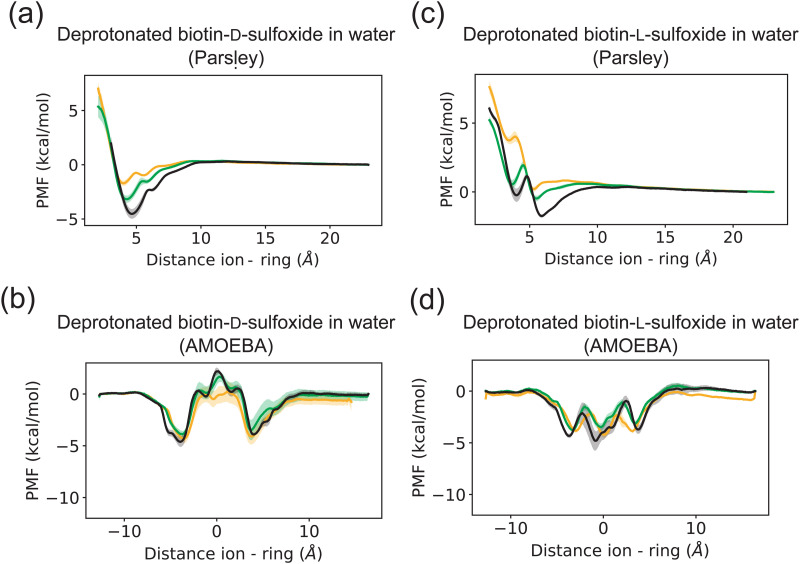
Potential of mean force (PMF) of ions and a protonated biotin sulfoxide macrocycle in two different conformations (d-conformer in (a and b), l-conformer in (c) and (d)) with unmethylated side chains in water for two different force fields (Parsley in the first row, AMOEBA in the second row). Each PMF here corresponds to the average of three data sets. The standard deviation of the three data sets is plotted as error bar. Chloride is depicted in yellow, bromide in green and iodide in black.

We therefore calculated the PMF for both sulfoxide isomers solvated in water. Andersen *et al.* could not unambiguously verify the stereochemistry of the two sulfoxide macrocycles, but argued that the hexasulfoxide used in their ITC study was the l-isomer.^8^ The oxidized macrocycle has the same selectivity trend in water as the methylated biotin[6]uril macrocycle *i.e.* larger ions are preferred over smaller ions. We could reproduce this selectivity trend with Parsley. The binding free energies corresponding to the PMFs in Fig. 5 and 6 are shown in Table 3. The halide anions bind better to biotin-d-sulfoxide than to l-sulfoxide and this likely arises from the negatively charged oxygen pointing slightly towards the cavity in the l-isomer thus creating an unfavorable electrostatic interaction between the anion and the macrocycle.

**Table 3:** Estimates for the binding free energies for biotin-sulfoxide[6]uril hexaacid ester and different anions in water. Binding free energies are estimated by the difference between minimum and bulk PMF in kcal mol^−1^. The PMF minima are corrected by eqn (3) to take the orthogonal degrees of freedom into account (note the binding energy is positive for bromide and the deprotonated host when using the Parsley force field)

Conformation	kcal mol^−1^	Chloride	Bromide	Iodide
l-Isomer (deprotonated)	ITC in water	/	−2.2	−3.8
	PMF (Parsley)	Unstable	+0.8 ± 0.2	−0.5 ± 0.1
	PMF (AMOEBA)	−2.9 ± 0.3	−2.5 ± 0.2	−3.5 ± 0.9
d-Isomer (deprotonated)	PMF (Parsley)	−0.4 ± 0.1	−1.9 ± 0.1	−3.2 ± 0.2
	PMF (AMOEBA)	−3.0 ± 0.4	−2.6 ± 0.3	−3.3 ± 0.5
l-Isomer (protonated)	PMF (Parsley)	Unstable	+0.7 ± 0.1	−1.1 ± 0.1
	PMF (AMOEBA)	−2.7 ± 0.7	−4.0 ± 0.4	−3.9 ± 0.5
d-Isomer (protonated)	PMF (Parsley)	−2.3 ± 0.1	−3.2 ± 0.1	−4.6 ± 0.2
	PMF (AMOEBA)	−4.3 ± 0.5	−3.9 ± 0.2	−3.7 ± 0.3

The selectivity trend in water could not be reproduced with AMOEBA and the binding free energies for the halide anions are within error (Table 3). There are two stable binding poses for anions with biotin-l-sulfoxide; the central one in the cavity and the second 3.5–4 Å from the centre of the cavity. For the d-isomer, only a non-central binding pose is stable and the halide anions cannot stably bind to the cavity centre.

### Comparison of the three macrocycles

**Fig. 7:**
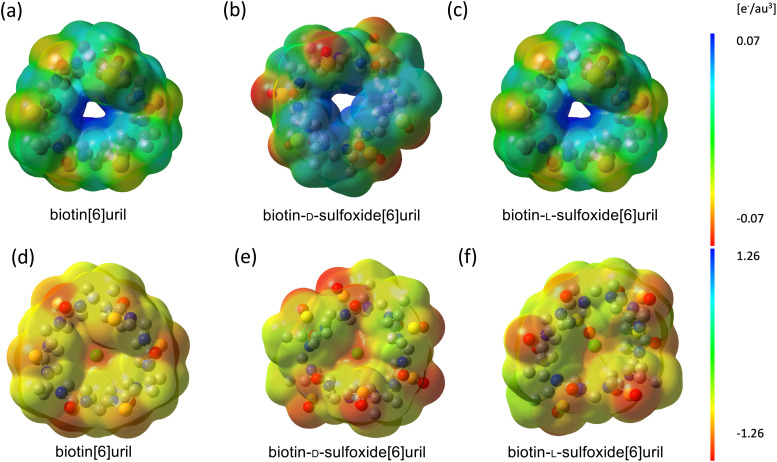
Electrostatic surface potentials of (a) biotin[6]uril, (b) biotin-d-sulfoxide[6]uril and (c) biotin-l-sulfoxide[6]uril in the apo state and (d–f) with chloride bound in the central cavity modelled at B3LYP/cc-pvtz level of theory. The macrocycles are shown top-down (as drawn in Fig. 1) looking into the cavities. The side chains have been truncated. Red, green and blue correspond to negative, neutral and positive potentials respectively. The cavity potential for the sulfide and d-sulfoxide macrocycle corresponds to approximately +130 kJ mol^−1^ whereas the l-sulfoxide isomer has a more positive cavity potential.

In general, the sulfide macrocycle is a “better host” than the sulfoxide macrocycle, which is a trend confirmed both by ITC and molecular dynamics simulations with and without polarizable force fields. The sulfoxide moiety is more electron withdrawing than sulfide. Hence, the sulfoxide macrocycles exhibit different electrostatic surface potentials. Andersen *et al.* calculated the electrostatic surface potential at the CAM-B3LYP/6-311G(d) level of theory.^8^ The cavity potential of biotin[6]uril and biotin-d-sulfoxide[6]uril corresponds to a surface potential of approximately +130 kJ mol^−1^. The l-sulfoxide conformer has a more positive surface potential of approximately +200 kJ mol^−1^ (see Fig. 7 for a B3LYP/cc-pvtz level of theory, which has been used for electrostatic fitting of monopole, dipoles and quadrupoles AMOEBA parameters). The cavity is neutral (green) for all three systems. The oxidized carriers are more polar either side of the cavity due to the negatively (red) charged additional oxygen.

Polarizable and nonpolarizable force fields predict different binding poses for the biotin[6]uril macrocycle and ions: in nonpolarizable force fields, halide anions cannot stably bind central in the hydrophobic cavity. The nonpolarizable binding pose that is 3.5–4 Å either side of the cavity does not correspond to a free energy minimum in simulations with the polarizable AMOEBA force fields, (as shown in Fig. 2).

Oxidizing the sulfide moiety makes the system more polar. We find the same stable binding poses in simulations with non-polarizable and with polarizable force fields. The L- and d-isomer have a free energy minimum around 3.5–4 Å either side of the cavity for both force field types. This non-central binding pose (Fig. 5) is stable both in AMOEBA and Parsley simulations. For the biotin-l-sulfoxide macrocycle, the polarizable AMOEBA force field reveals another stable binding pose not seen in non-polarizable simulations. The central binding pose is stable only for the biotin-l-sulfoxide (and of the biotin[6]uril) macrocycle in the AMOEBA framework.

### Comparison of the Parsley and AMOEBA force fields

We examined the conformational ensembles and host flexibilities and rigidities of biotin and biotin sulfide using both the AMOEBA and Parsley force fields. We compared the RMSD values of the twelve cavity carbon atoms between the two force fields (Fig. S4, ESI†) and found that the average RMSD was smaller than 1 Å. To further characterize the conformational behaviour, we analysed the C–N–C–N dihedral distributions, which describe the orientation of two subunits to each other. The distributions were similar across all systems and force fields (Fig. S5, ESI†). Our results suggest that the behaviour of the hosts is consistent in both force fields, indicating that the biotin[6]uril and biotin-sulfoxide macrocycles have similar flexibilities and rigidities across different force fields.

At this juncture, it is worth noting that different van der Waals interaction potentials can give rise to different radii (Fig. S1, ESI†). To test whether the difference in radius contributed to the lack of binding of the chloride anion to the centre of the cavity, we fitted the Lennard-Jones potential to the buffered 14-7 potential (see Fig. S6, ESI†). We also used the chloride parameters from Zhang *et al.*^22^ For both of these additional parameterizations, we found that chloride was not stably bound centrally in the cavity. In addition, fluoride, which has a smaller radius than chloride, was also not stable in the cavity (see Fig. S6(c), ESI† for a PMF for fluoride). Thus, taken together these results suggest that a smaller ion is not stable inside the centre of the cavity and that the instability of chloride is not attributable simply to the use of the 12-6 potential from Li *et al.*^14^ Thus, although Parsley captures the trends observed in ITC, it does not capture the observed binding mode. AMOEBA on the other hand does capture the binding mode but is less able to capture the observed trends. We thus explored the host-ion energetics in more detail.

### Interaction energies between hosts and ions

As AMOEBA captures the binding mode, we took conformations from these simulations and the computed the interaction energies between the chloride guest and the three different hosts using both force fields. These windows consisted of 13 frames selected from AMOEBA simulations that spanned 13 ns, with each frame being 1 ns apart. By calculating the interaction energy profiles along the reaction coordinates, we were able to decompose the total energy into its individual components. The interaction energy profiles along the reaction coordinate are computed with respect to bulk as the reference point: the interaction-energy of host and guest is set to be zero for a distance of 15 Å from the centre of the cavity.

**Fig. 8:**
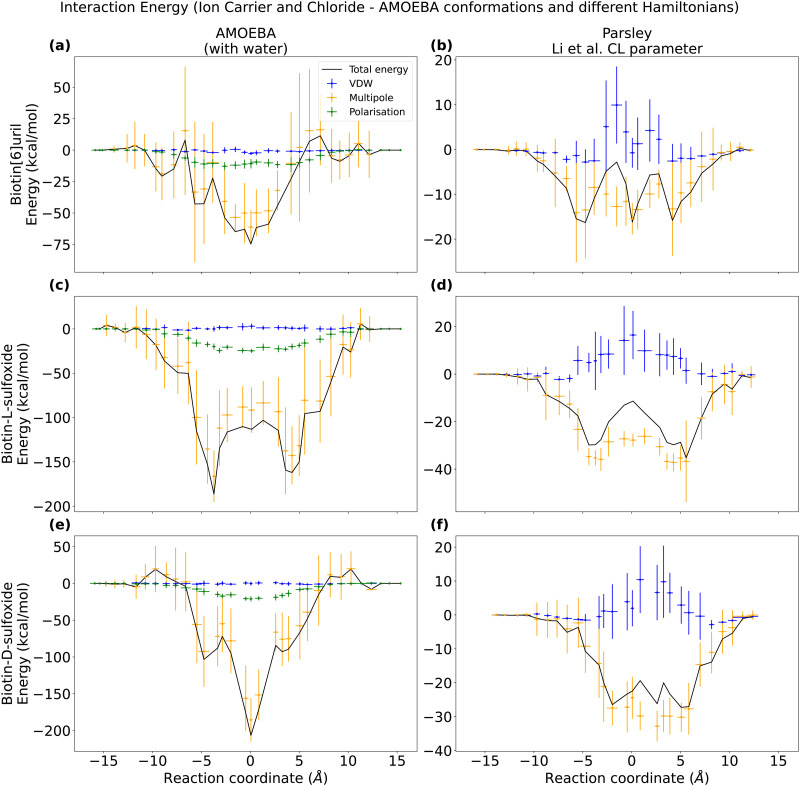
Interaction energies along the reaction coordinate for three ion carriers and chloride in water evaluated from the AMOEBA conformations but calculated with either the AMOEBA (a, c and e) or the Parsley (b, d and f) force field. For the Parsley simulations, interactions are computed for vdw (blue) and electrostatics (yellow). For each 13 ns long umbrella window, 13 conformations were selected to evaluate the interaction energy. Note the different energy scales.

To assess the pairwise contribution to the interaction energies using the AMOEBA parameters, we utilized the Tinker ANALYZE program. Since polarizable force fields are nonlinear, it was necessary to account for multi-body contributions. Therefore, we evaluated the interaction energies both in the presence and absence of solvent to capture these effects. The total interaction energy between the chloride guest and the host differs by a few kcal mol^−1^ when the water is present or not when calculating the energies (Fig. S7, ESI†), but the overall trend along the reaction coordinate is preserved and minima in the PMF correspond to minima of the interaction energy (Fig. 8).

In order to evaluate the interaction energies using the Parsley force field in GROMACS, we converted the frames obtained from the AMOEBA simulation to GROMACS format (via MDAnalysis).^39^ We evaluated the AMOEBA frames with the Parsley force field for two different chloride parameter sets: (1) Li *et al.*^14^ parameter set (with a VDW radius larger than the one in AMOEBA, see Fig. 8). (2) Re-fitted VDW parameters that resemble the VDW chloride radius in AMOEBA (Fig. S6, ESI†). When evaluating the interaction energies of the conformations sampled in AMOEBA simulations with the Parsley force field (Fig. S8, ESI†), we observe that the Lennard-Jones potential is repulsive close to the cavity when using the Li *et al.*^14^ parameter set for chloride. However, when we use the re-fitted parameters (Fig. S6, ESI†), the repulsive VDW contribution vanishes. We also evaluated the interaction energies along the reaction coordinate for the Parsley umbrella simulations with the Parsley force field (Fig. S9, ESI†). Even though the central binding pose is not stable, and the potential of mean force has no minimum (Fig. 4 and 6), the interaction energy is minimal close to the cavity centre. The VDW contribution are not repulsive close to the cavity when we evaluate the Parsley derived conformations. Therefore, we conclude that the difference in binding behaviour is not due to the difference in VDW parameters for the ions. Ions with smaller VDW radius than the Li *et al.*^14^ chloride are not stably bound at all in the cavity (Fig. S6, ESI†).

**Fig. 9:**
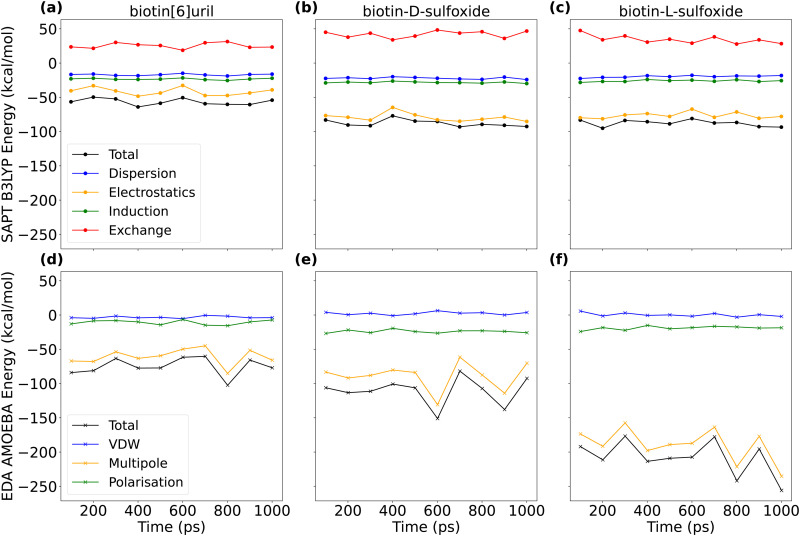
Interaction energies between chloride and three hosts (biotin[6]uril, biotin-d-sulfoxide[6]uril and biotin-L-sulfoxide[6]uril). For ten AMOEBA frames, we evaluated the interaction energy with symmetry-adapted perturbation theory (SAPT) on B3LYP level of theory with a jun-cc-pVDZ basis set (a–c) and performed interaction energy decomposition analysis (EDA) with the AMOEBA force field (d–f). Mean and standard deviation of the energy components are summarized in Tables S2 and S3 (ESI†). Interaction energies for the non-central binding pose are plotted in Fig. S10 (ESI†).

After having compared interaction energies for a non-polarizable and a polarizable force field, we performed symmetry-adapted perturbation theory (SAPT) at the B3LYP level of theory to obtain a “ground truth” for the interaction energy between the hosts and chloride. We used the jun-cc-pVDZ basis set, which includes chloride. We extracted ten AMOEBA frames and then compared the energy decomposition from density functional theory (DFT) with an equivalent energy decomposition from the AMOEBA force field (Fig. 9 and Tables S2, S3, ESI†). In the AMOEBA force field, there is no exchange term (red in Fig. 9) and the van der Waals term is less favorable than the dispersion term.

Comparing the interaction energies for the Parsley and AMOEBA force field with the DFT energy decomposition highlights the fact that host systems with narrow and hydrophobic cavities cannot be accurately described with conventional additive force fields such as Parsley that are missing the higher multipoles and polarization. AMOEBA does not incorporate the exchange term, and this is non-negligible. However, AMOEBA represents a good trade-off in terms of accuracy, sampling and performance. The AMOEBA multipole interaction energy for the l-sulfoxide macrocycle exceeds the one for the d-sulfoxide macrocycle (Fig. 9 and Table S3, ESI†). The oxygen in the sulfoxide group is closer to the chloride bound centrally in the cavity in the d-sulfoxide than in the l-sulfoxide macrocycle. The multipole interaction between these oxygens and chloride are repulsive. In the central binding pose, the interaction energy in the l-sulfoxide system is larger because the non-favourable CL–O interaction is weaker (see Fig. S11, ESI†).

### Role of water in interactions of anions with hosts

**Fig. 10:**
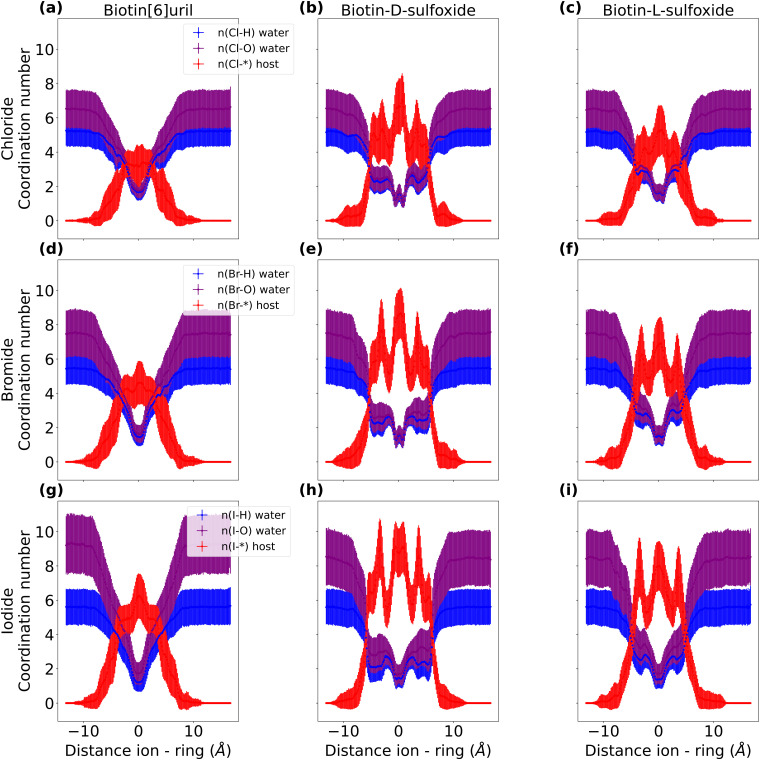
Hydration shell of halide anions in biotin[6]uril (a–c), biotin-d-sulfoxide (d–f) and biotin-l-sulfoxide (g–i) in water (AMOEBA). For each time-step in each umbrella window, the number of interactions within a cut-off are counted. The interactions and the distance of the ion to the biotin ring are stored for each time-step and binned. The error bars correspond to the standard deviation of each bin.

Given the performance of AMOEBA thus far, we therefore also investigated the role of waters in the interactions of anions with biotin[6]uril, biotin-d-sulfoxide and biotin-l-sulfoxide. The behaviour of the hydration shells as a function of the distance between ion and cavity center with the AMOEBA force field is summarized in Fig. 10 and Table S4, Fig. S12, ESI†). There are more ion interactions with the cavity of the sulfoxide macrocycles than with the sulfide moiety. The hydration shell of each ion is partially replaced with cavity interactions in all three systems when the ion binds centrally to the cavity. However, the hydration shell is replaced to a larger extent in the sulfoxide macrocycles. Furthermore, ions start replacing their hydration shell earlier with interactions with the sulfoxide host: the hydration shell is almost intact for an ion that is 4 Å away from the cavity center of the macrocycle with the sulfide moiety. This non-central binding pose is not stable and does not correspond to a PMF minimum. However, the hydration shell is already partially replaced with interactions with the sulfoxide macrocycle at a distance of 4 Å and this non-central binding pose is stable and corresponds to a PMF minimum for both sulfoxide isomers.

Furthermore, we compared the interaction energy between chloride and the biotin[6]uril macrocycle and a water molecule bound in the cavity and the macrocycle. As expected, there is a stronger polarization interaction energy between chloride and host than between water and host (Fig. S13, ESI†). The guests are both centrally bound in the cavity.

**Fig. 11:**
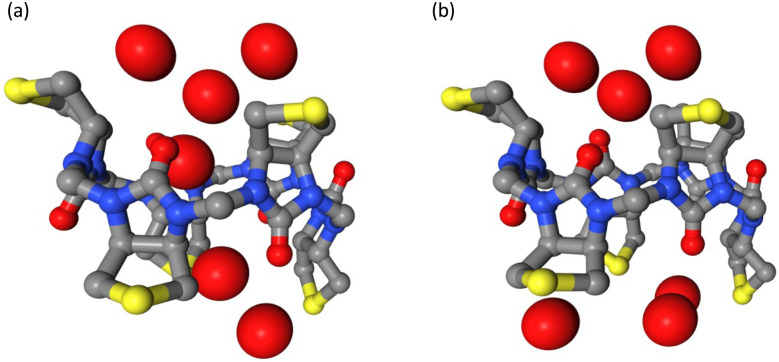
Observed hydration patterns: (a) wetted cavity and (b) de-wetted cavity of biotin-sulfide macrocycle. The side chains of the biotin units and hydrogens are not shown.

The cavities of the different macrocycles differ in their electrostatic surface potential (see Fig. 7) and this influences the behaviour of water in the confined environment of the cavity. We observed two different states; either the cavity is hydrated (water molecules are within 3 Å of the cavity center, Fig. 11a) or it is de-wetted (no water molecule within the cavity, Fig. 11b). In the macrocycles with less polar cavities, the cavity is de-wetted during 10–15% of the simulation time. In the sulfide macrocycle, the transitions occur more often and are shorter lived than in d-sulfoxide. In the more polar l-sulfoxide macrocycle, the cavity is de-wetted in only 1% of the simulation time. An ion entering the cavity of the l-sulfoxide competes with what appears to be a relatively stably bound water molecule. This explains the weaker binding affinity of the l-sulfoxide host compared to the d-sulfoxide and sulfide host. In simulations employing Parsley, the cavities of all three hosts are de-wetted in less than 0.1% of the simulation time. It is clear that the different behaviour of water in a confined environment with varying polarity can therefore only be accurately simulated with a polarizable force field.

De-wetting transitions can be also observed in narrow and hydrophobic ion channels^38^ but occur on a nanosecond time scale. The de-wetting transition in the smaller anion carriers occurs on a picosecond time scale and is more dynamic than a de-wetted state in a larger ion channel with a similar radius of about 3 Å. In ion channels, the de-wetted state can be de-stabilized by changing the polarity of the pore lining.^38^ The cavity of the macrocycle is therefore more likely to be hydrated in the polar l-sulfoxide version, whereas in the sulfide macrocycle with a nonpolar cavity, the de-wetting transition occurs more frequently (Fig. S14, ESI†).

## 4. Conclusions

In this study, binding free energies have been calculated for biotin macrocycles and different anions in water and acetonitrile with *in silico* methods employing both non-polarizable and polarizable force fields. The anion binding showed strong solvent dependency, which is consistent with results from experimental studies.^6,8^ However, the free energy profiles obtained by PMF and the preferred binding positions of anions were found to depend on whether the force field can model the effect of multipoles and induced polarization.

Lisbjerg *et al.* reported that anion binding in the biotin[6]uril family is solvent dependent.^6^ Biotin[6]uril hexa-acid preferentially binds larger anions in water while biotin[6]uril hexamethyl ester binds smaller anions in organic solvents such as acetonitrile and can transport anions across cell membranes.^8^ The influence of solvent therefore has direct functional implications: Biotin[6]uril hexamethyl ester will bind anions strongly when it is inside a low dielectric lipid membrane environment for transport but binds weakly at a high dielectric lipid–water interface for spontaneous ion uptake and release. It also binds chloride stronger in a nonpolar solvent than in water. Confirming the selectivity trend in two different solvents with different force fields is therefore encouraging. However, the preferred binding pose and the magnitude of the binding affinity is highly dependent on the force field type. Simulations with the polarizable force field AMOEBA overestimate the binding free energies as summarized in Tables 1 and 2. The cavity of the anionophore is lined by 12 nonpolar CH-groups and is reportedly hydrophobic.^6^ The interactions of anions with soft hydrogen bond donors in the cavity depends on the force field and of course the partial charges of the CH-groups in the cavity are fixed when using Parsley. In simulations with non-polarizable force fields, ions are not stably bound in the nonpolar cavity. They prefer configurations where the ion is 3.5–4 Å from the centre of the ring, where they interact with a more polar environment as depicted in Fig. 2. The inclusion of multipoles and polarization allows the AMOEBA force field to respond to changing heterogeneous molecular environments. The binding pose from AMOEBA simulations (Fig. 3a) resembles the one reported in the crystal structure^7^ and the ions are encapsulated in the slightly hydrophobic cavity.

Parametrization processes can sometimes result in different effective radii. When comparing the chloride VDW potential used in the Parsley and AMOEBA simulations in Fig. S1c (ESI†), it is apparent that the Li *et al.* VDW radius is larger than the AMOEBA VDW radius, thus potentially explaining why chloride in non-polarizable force fields may prefer not to sit within the centre of the cavity. However, when we decrease the VDW radius of chloride in the Parsley simulations, we still do not observe any stable binding pose in the cavity. Interestingly, even fluoride, which has a smaller radius than chloride, was also unable to form a stable binding pose in the cavity, suggesting that the instability was not due to the size of the ion.

The cavity of both isomers of the biotin-sulfoxide macrocycle is more polar than the biotin[6]uril cavity. The difference of the free energy landscapes of the more polar macrocycles for non-polarizable and polarizable force fields are less pronounced than for the more hydrophobic biotin[6]uril. The different force field frameworks predict completely different binding poses for the more hydrophobic biotin[6]uril macrocycle, whereas the polarizable AMOEBA force field predicts the same binding pose for biotin-sulfoxide as the non-polarizable Parsley force field. In case of the l-isomer, the polarizable simulations indicate another binding pose centrally in the cavity that is unstable in simulations with the Parsley force field.

Polarizable force fields are computationally more expensive than pairwise additive force fields (see Table S5, ESI†), but non-polarizable force fields cannot capture the effect of induced polarization where the electronic structure of an atom is altered by the varying distribution of charges in its environment. In conclusion, this study highlights the sensitivity of binding free energies and binding positions to the choice of force fields. AMOEBA captures the binding poses well, but reproducing energetic trends, at least in these systems, requires further work. Ion binding in a nonpolar environment with soft hydrogen-bond donors therefore represents a useful case study where the higher computational costs of employing a polarizable framework are justified and yield a more accurate description than pairwise additive force fields. In addition, the ease of performing these kinds of calculations will become easier as certain parts of the process become more automated.^40^ In general, these results not only have important implications for our understanding of anion host interactions in synthetic ionophores, but also in narrow cavities of biological ion channels.

## Data availability

Force field and simulation parameters and simulation boxes are available on https://github.com/DSeiferth/anion-polarization and forked at https://github.com/bigginlab/anion-polarization.

## Author contributions

PCB and DS conceived the project. DS performed and analysed all simulations. DS wrote the first draft and all authors contributed to revising and editing the manuscript.

## Conflicts of interest

There are no conflicts to declare.

## Supplementary Material

CP-025-D3CP00479A-s001
