# Correction: Limitations of non-polarizable force fields in describing anion binding poses in non-polar synthetic hosts

**DOI:** 10.1039/d3cp90150e

**Published:** 2023-07-17

**Authors:** David Seiferth, Stephen J. Tucker, Philip C. Biggin

**Affiliations:** a Clarendon Laboratory, Department of Physics, University of Oxford Oxford OX1 3PU UK; b Structural Bioinformatics and Computational Biochemistry, Department of Biochemistry, University of Oxford Oxford OX1 3QU UK philip.biggin@bioch.ox.ac.uk; c Kavli Institute for Nanoscience Discovery, University of Oxford Oxford UK

## Abstract

Correction for ‘Limitations of non-polarizable force fields in describing anion binding poses in non-polar synthetic hosts’ by David Seiferth *et al.*, *Phys. Chem. Chem. Phys.*, 2023, **25**, 17596–17608, https://doi.org/10.1039/D3CP00479A.

Fig. 6 in the published version of the article was incorrect as it was a copy of Fig. 5. The correct figure is given below.

**Fig. 6:**
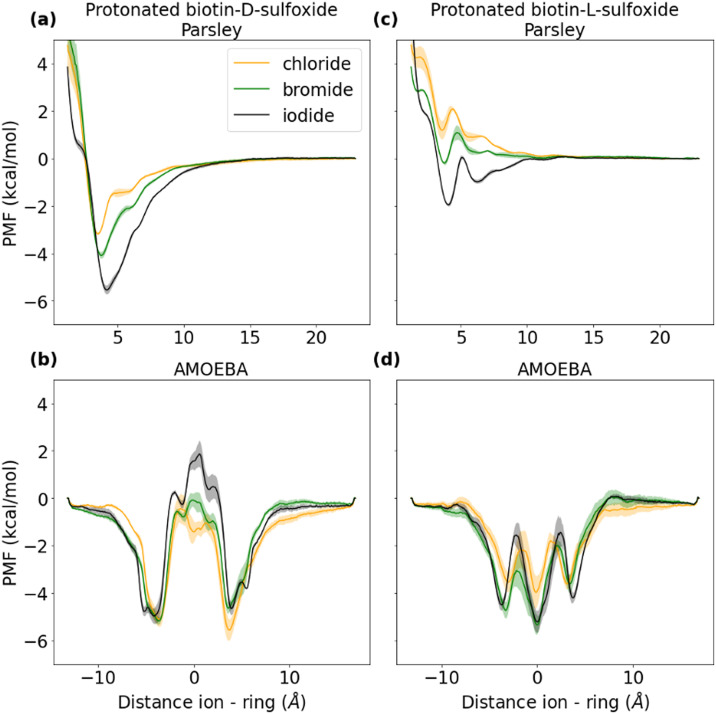
Potential of mean force (PMF) of ions and a protonated biotin sulfoxide macrocycle in two different conformations (d-conformer in (a and b), l-conformer in (c) and (d)) with unmethylated side chains in water for two different force fields (Parsley in the first row, AMOEBA in the second row). Each PMF here corresponds to the average of three data sets. The standard deviation of the three data sets is plotted as error bar. Chloride is depicted in yellow, bromide in green and iodide in black.

The Royal Society of Chemistry apologises for these errors and any consequent inconvenience to authors and readers.

## Supplementary Material

