# Supplementary material for: Limitations of non-polarizable force fields in describing anion binding poses in non-polar synthetic hosts
Source: Phys Chem Chem Phys. 2023 Jun 20;25(26):17596–608. doi: 10.1039/d3cp00479a (PMC10321056; doi:10.1039/d3cp00479a)
Supplement: CP-025-D3CP00479A-s001 [file CP-025-D3CP00479A-s001.pdf]

Electronic Supplementary Material (ESI) for Physical Chemistry Chemical Physics.
This journal is © the Owner Societies 2023

SUPPLEMENTARY INFORMATION

Limitations of Non-Polarizable Force Fields in Describing Anion Binding Poses in Non-Polar Synthetic Hosts

David Seiferth[1,2], Stephen J. Tucker[1,3] and Philip C. Biggin[2]*

SI Text 1

Force field Parametrization for Biotin Macrocycles

For non-polarizable, pairwise additive force fields, parameters for the methylated and unmethylated biotin macrocycles were generated with the second generation of General AMBER Force Field (GAFF2)[1] and the Parsley force field from the Open Force Field Initiative.[2] To assign force field parameters to biotin[6]uril hexamethyl ester, we used antechamber and tleap for the GAFF2 force field.[3] For the non-bonded interactions, van der Waals parameters are inherited from the AMBER force fields and partial charges are assigned according to the so-called Austin Model 1 bond charge corrections (AM1-BCC) model. Mulliken charges are obtained by a semi-empirical AM1 calculation, followed by a bond charge correction (BCC) scheme.[1] The Parsley force field relies on an AM1-BCC model for charge assignment as well[2]. Simulations with pairwise additive force fields (GAFF2 and Parsley) were carried out using the three sites transferable intermolecular potential (TIP3P) water model[4]. We used the parameters for halide ions from Li et al. and other parameters for non-bonded interactions were taken from the AMBER force field.[5, 6]

The AMOEBA force field (Atomic Multipole Optimized Energetics for Biomolecular Applications) incorporates a polarizable multipole framework up to quadrupole moments.[7] We used the AMOEBA03 parameters for water.[8] For the parameters for monovalent ions (halide anions, and potassium) and acetonitrile, we used the AMOEBA09 parameter set.[7] The parametrization of biotin[6]uril hexamethyl ester for the AMOEBA force field followed the POLTYPE protocol.[9] We used density functional theory calculations to derive electrostatic parameters. The defaults in the POLTYPE protocol are *ab initio* calculations which were also used by Laury *et al*. for the parametrization of the cucurbit[8]uril molecule.[10] The initial structure was optimized using the semi-empirical PM3 method and then optimized with B3LYP level of theory with a 6-311G(1d,1p) basis set and empirical dispersion gdBJ using the Gaussian 16 software package. The optimized structure was used as an input for a distributed multipole analysis using Stone's GDMA[11] that determined initial atomic multipole estimates such as the charge, the

components of the dipole vector and the quadrupole tensor. The Tinker program POLEDIT was used to choose local multipole frames and atomic polarizabilities and to define polarization groups. A polarization group in AMOEBA defines a group of atoms whose permanent multipoles do not polarize one another. In AMOEBA, the multipole moment vector is decomposed into a permanent contribution, independent of the environment and an induced component that can change according to the local electric field of other atoms. Within a polarization group, the permanent multipoles are excluded from contributing to the field at an atomic site, and hence only the other induced dipoles polarize the atom in question.[12] A single point calculation at B3LYP level of theory with cc-ptvz basis and empirical dispersion gd3BJ was used as a reference point for electrostatic potential fitting. A Cartesian grid of points, at which to calculate the electrostatic potential, was created with the tinker POTENTIAL program.[13] The quantum mechanical (QM) electrostatic potential was evaluated at each of these grid points with the Gaussian CUBEGEN program. We then evaluated the AMOEBA electrostatic potential at each grid point and fitted the dipole and quadrupole components to minimize the root mean square error between the QM and AMOEBA electrostatic potential with the tinker POTENTIAL program while keeping the partial charges (the monopoles) fixed.[13] Finally, we assigned parameters for bonds, angles, stretch-bends, out-of-plane bends, torsions and van der Waals interactions by similarity to the AMOEBA09 parameter set with the tinker VALENCE program.[7, 13] The AMOEBA09 parameter file contains atom classes for many functional groups such as saturated carbon and hydrogen, amine nitrogen, sulfides and carboxylic acids.

Ion Parameters

Within molecular mechanics force-fields, the 12-6 Lennard-Jones potential is the most commonly used potential to model van der Waals interactions. The repulsive term is proportional to r^{-12} and the attractive term to r^{-6}:

$$U_{VDW} = 4\varepsilon_{ij}\left(\left(\frac{\sigma_{ij}}{r_{ij}}\right)^{12} - \left(\frac{\sigma_{ij}}{r_{ij}}\right)^{6}\right) \qquad (1)$$

where σ refers to the zero of the potential and ε to the well depth of its minimum.

Each atom *i* is characterized by a parameter pair σ_i, ε_i. The pairwise interaction between atom *i* and *j* can be modeled with Lorentz-Berthelot combination rules for radius and energy:

$$\sigma_{ij} = \frac{\sigma_i + \sigma_j}{2} \qquad (2)$$

$$\varepsilon_{ij} = \sqrt{\varepsilon_i \epsilon_j} \qquad (3)$$

In simulations with Parsley[2], which we selected as the non-polarizable force-field, we used the ion parameters from Li *et al.*, which were parameterized to reproduce hydration free energies.[6] **Fig. S1a** shows the 12-6 Lennard-Jones potential for the four halide anions.

With increasing ion size, both σ and ε increase. In simulations with ion parameters from the AMBER03 parameter set, the ions are not stably bound inside the cavity and this likely reflects the fact that the AMBER03 Lennard-Jones potential for halide anions is much shallower (**Fig. S1c**) compared to the other parameter sets considered here.

To test whether the difference in radius contributed to the lack of binding of the chloride anion to the centre of the cavity, we fitted the Lennard-Jones potential to the buffered 14-7 potential in Fig. S4. The Lennard-Jones-fit resembling the AMOEBA buffered 14-7 potential does not preserve the hydration free energy of chloride in water. The chloride with fitted LJ parameters is not stably bound in the cavities of

the macrocycles. We also used the chloride parameters from Zhang et al. 202114 and found that chloride was not stably bound centrally in the cavity either. The Zhang et al. parameter set 14 was recently commented on in a study by Smith *et al.*[15] in which they warn about overfitting the van der Waals radii of ions. The chloride parameters from Li *et al.*[6] relate the radius and the well depth via a so-called "noble-gas" curve which is a fit based on the VDW parameters of noble gas atoms to correlate the radius and the well depth in the LJ equation.

Many non-polarizable force fields are fitted to reproduce gas-phase conformational energies and geometries. Non-bonded interactions are particularly important in condensed phase simulations. Of the non-bonded interactions, electrostatics models are often polarized beyond what would be expected in the gas phase.[16] The transferability of parameters for pairwise additive force fields can be limited due to their fixed charges and their inability to model the effect of induced polarization. The Parsley force field uses a fixed partial charge model and a Coulomb potential to describe electrostatic interactions. A fixed partial charge model corresponds to a multipole expansion where dipole, quadrupole and higher-order terms are truncated.

Compared to additive force-fields, the AMOEBA force field[7, 17] uses different potentials for non-bonded interactions. Van der Waals interactions are described by a buffered 14-7 potential of the form

$$U_{VDW} = \varepsilon_{ij}\left(\frac{1+\delta}{\rho_{ij}+\delta}\right)^7\left(\frac{1+\gamma}{{\rho_{ij}}^7+\gamma}-2\right) \qquad (4)$$

With $\delta = 0.07$ and $\gamma = 0.12$ [17]. Here $\rho = r_{ij}/R_{ij}$ denotes the distance r_{ij} between two atoms *i* and *j* in units of the distance R_{ij} for which the potential is minimal. The depth of the potential well ε_{ij} and R_{ij} for atoms of different types are defined as

$$R_{ij} = \frac{R_i^3+R_j^3}{R_i^2+R_j^2} \qquad (5)$$

$$\varepsilon_{ij} = \frac{4\epsilon_i \varepsilon_j}{\left(\sqrt{\varepsilon_i}+\sqrt{\varepsilon_j}\right)^2} \quad (6)$$

The AMOEBA force field takes multipoles up to quadrupoles at each atomic centre into account and truncates octupole and higher moments. The electrostatic energy in AMOEBA includes contributions from both permanent and induced multipoles. Only the dipole moment is treated as inducible. Monopole (i.e. charge) and quadrupole moments are invariable. The inclusion of explicit dipole polarization allows the AMOEBA model to respond to changing or heterogeneous molecular environments.[17] An external field distorts the atomic electron density. A classical point dipole moment is induced at each polarizable atomic site according to the electric field felt by that site in AMOEBA. In order to prevent the divergence of induced dipole moments at short interatomic distances, the so-called polarization catastrophe, the AMOEBA force field incorporates Thole's damped interaction method.[18] Polarization interaction at very short range is damped by smearing out the atomic multipole moments. The charge density, ρ, in Thole's screening model is defined as:

$$\rho = \frac{3a}{4\pi} \exp\left(-a\, \tilde{r}_{ij}^3\right) \quad (7)$$

$$\tilde{r}_{ij} = \frac{r_{ij}}{\left(\alpha_i \alpha_j\right)^{1/6}} \quad (8)$$

Where $\tilde{r}_{ij}$ denotes the reduced interatomic distance depending on the atomic polarisabilities α_i. In the AMOEBA force field, all atoms have the same dimensionless width parameter $a = 0.39$ of the smeared charge distribution.

SI Text 2. Effects of Refitting Ion Parameters

The chloride with fitted LJ potential was placed centrally in the ring, energy minimized, and carefully equilibrated. 1ns NVT, 1ns NPT, 1ns NPT with restraints of 1500 and 1000 and 500 kJmol/nm^2 restraints respectively to ensure that the ion is centrally bound in the cavity. To allow flexibility in the xy-plane of the ring, we applied flat-bottom restraints similar in set-up as in the umbrella simulations. All restraints were lifted for production runs of 100 ns.

We set up three repeats with the set-up described above and in all three repeats, the chloride with modified VDW parameters left the cavity almost immediately. In the combined simulation time of 300 ns, we did not observe any re-binding event (Fig. S4b).

We also calculated the potential of mean force profile along the reaction coordinate for the chloride with modified VDW parameters and for fluoride, which has an even smaller VDW radius (Fig. S4c). Both ions cannot be stably bound by the biotin[6]uril host. Even though the interaction energies between host and ions with smaller VDW radius are favorable (Fig. S4d, e), the ions do not bind centrally to the cavity.

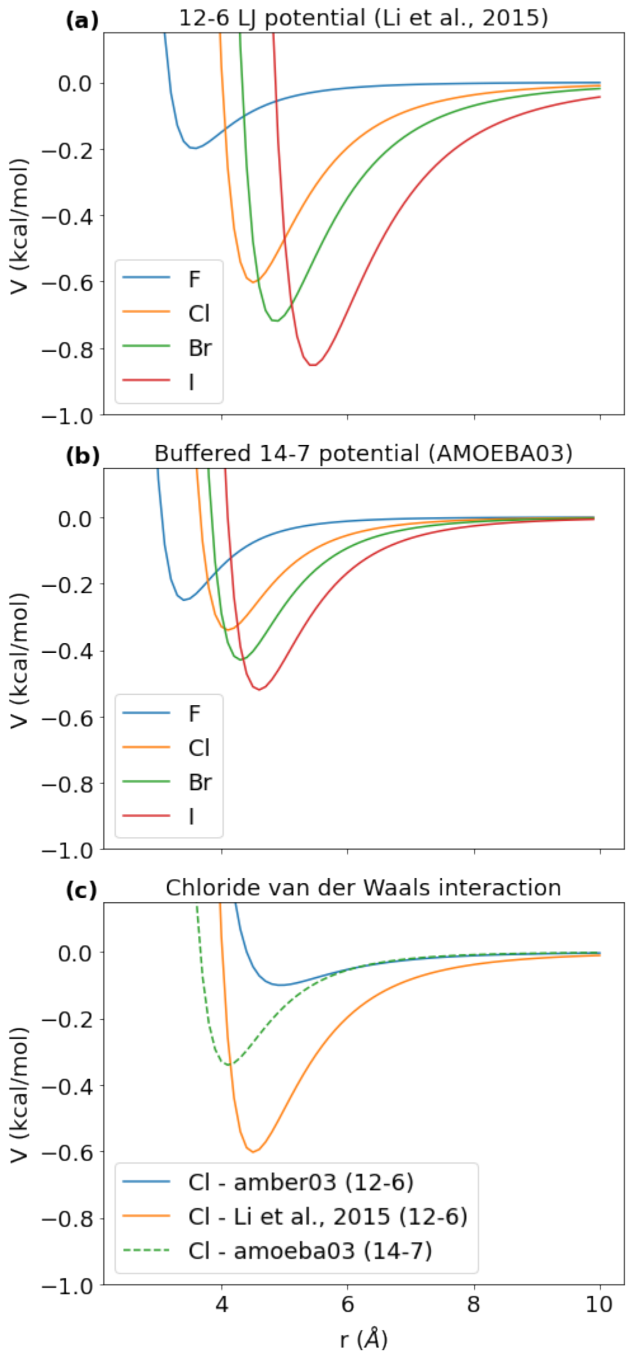

Figure S1. *van der Waals interaction modelled with a 12-6 Lennard-Jones potential (a) used for non-polarizable simulations and with a buffered 14-7 potential (b) used in AMOEBA. Different van der Waals models are compared in (c). In all plots, the van der Waals interactions are plotted for a pair of ions of the same type (fluoride – fluoride, chloride – chloride, etc).*

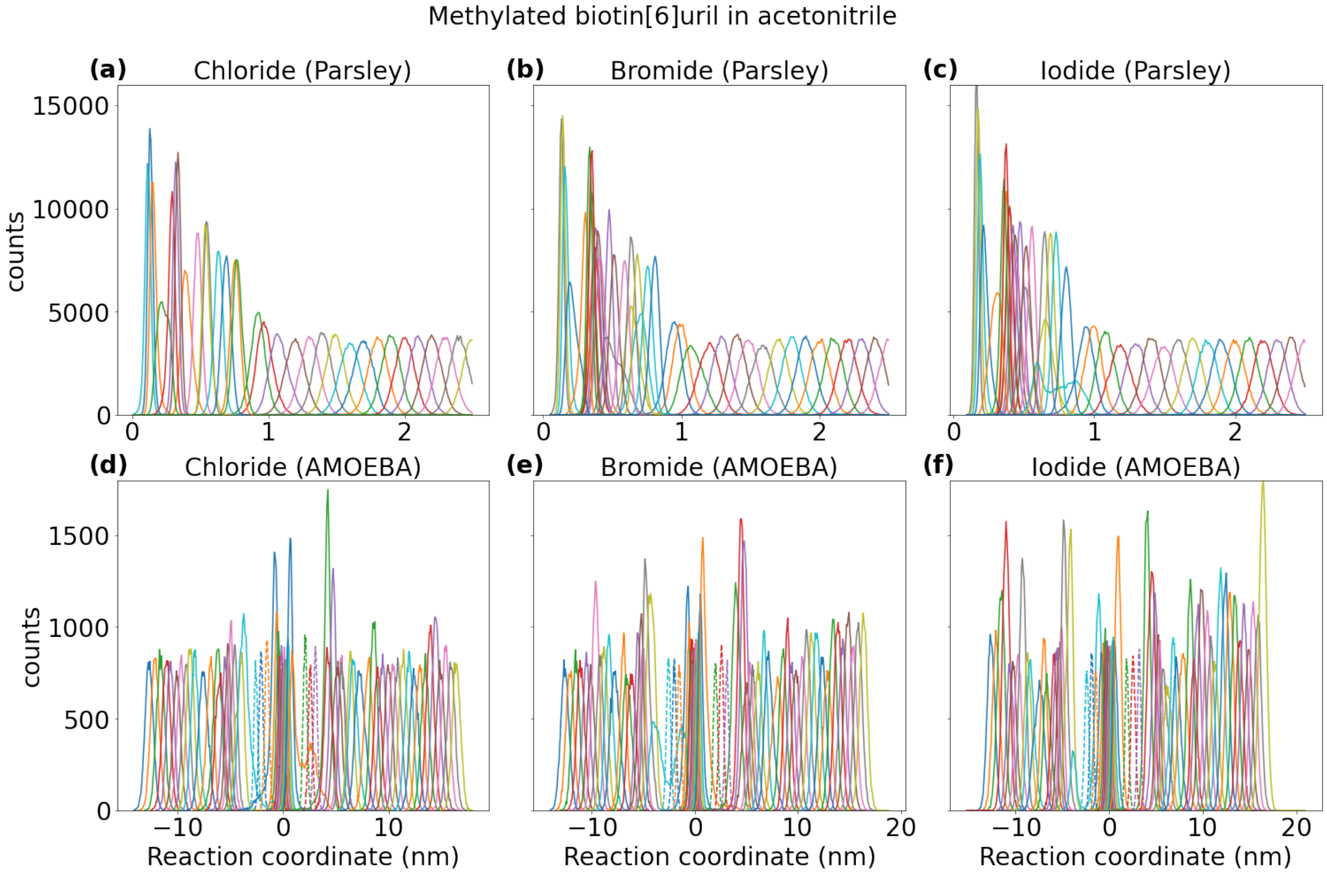

***Figure S2**. Histogram overlap for methylated biotin[6]uril in acetonitrile with Parsley (a, b and c) and with AMOEBA (d, e, f). The dashed lines indicate a higher force constant (10 kcal/mol/Å²) rather than 2 kcal/mol/Å².*

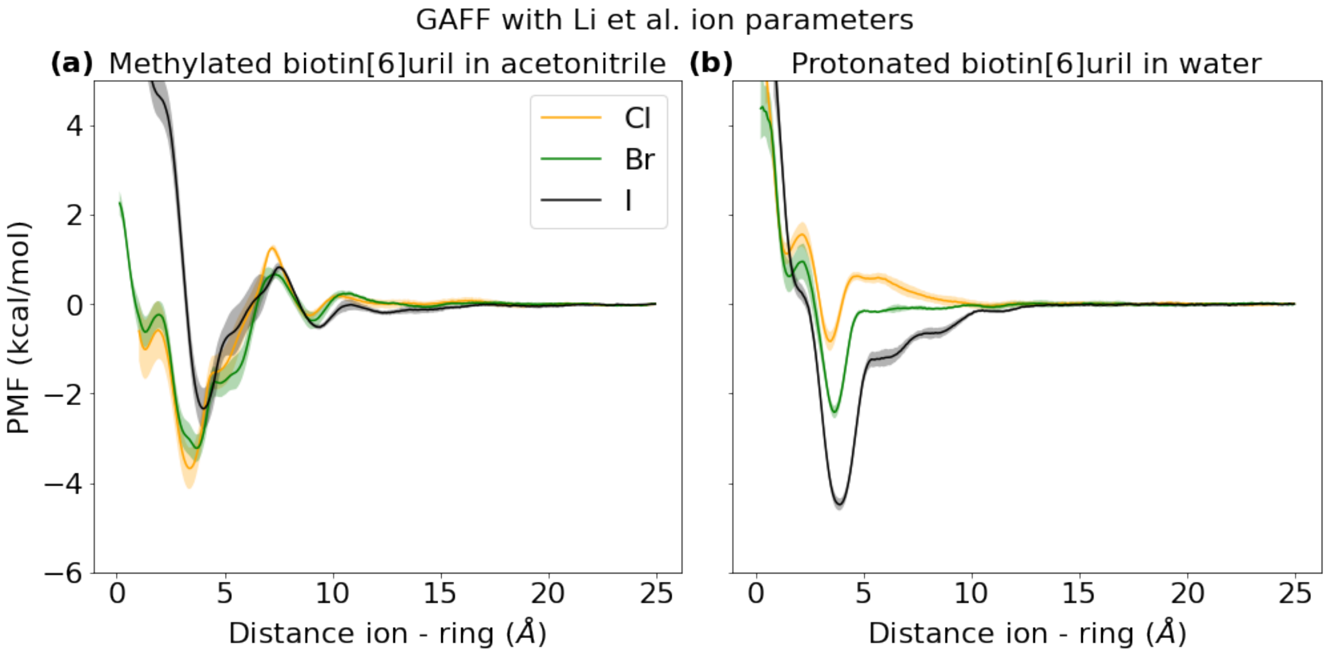

Figure S3. *Potential of mean force (PMF) of ions and a biotin macrocycle with methylated side chains in acetonitrile (a) and an unmethylated macrocycle in water (b) in protonated forms for two different force fields. Each PMF here corresponds to the average of three data sets. Error bars represent one standard deviation. The ions are not centrally bound in the cavity and the PMF profile look similar to those obtained with Parsley.*

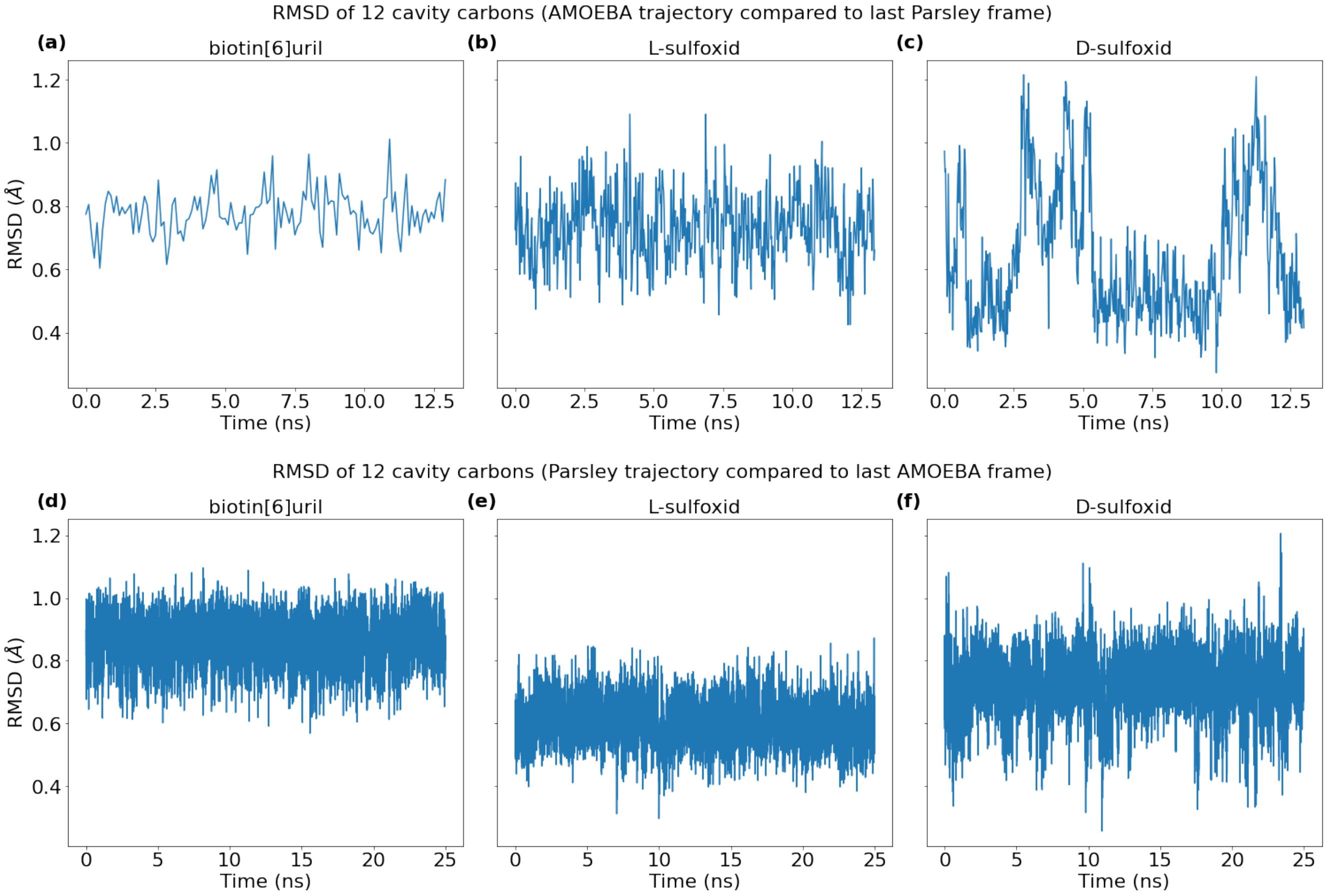

Figure S4. *(a)-(c) Time series of RMSD values comparing the AMOEBA trajectories to the last Parsley frame for three different biotin macrocycles in water. (b) Time series of RMSD values comparing the Parsley trajectories to the last AMOEBA frame for three different biotin macrocycles in water.*

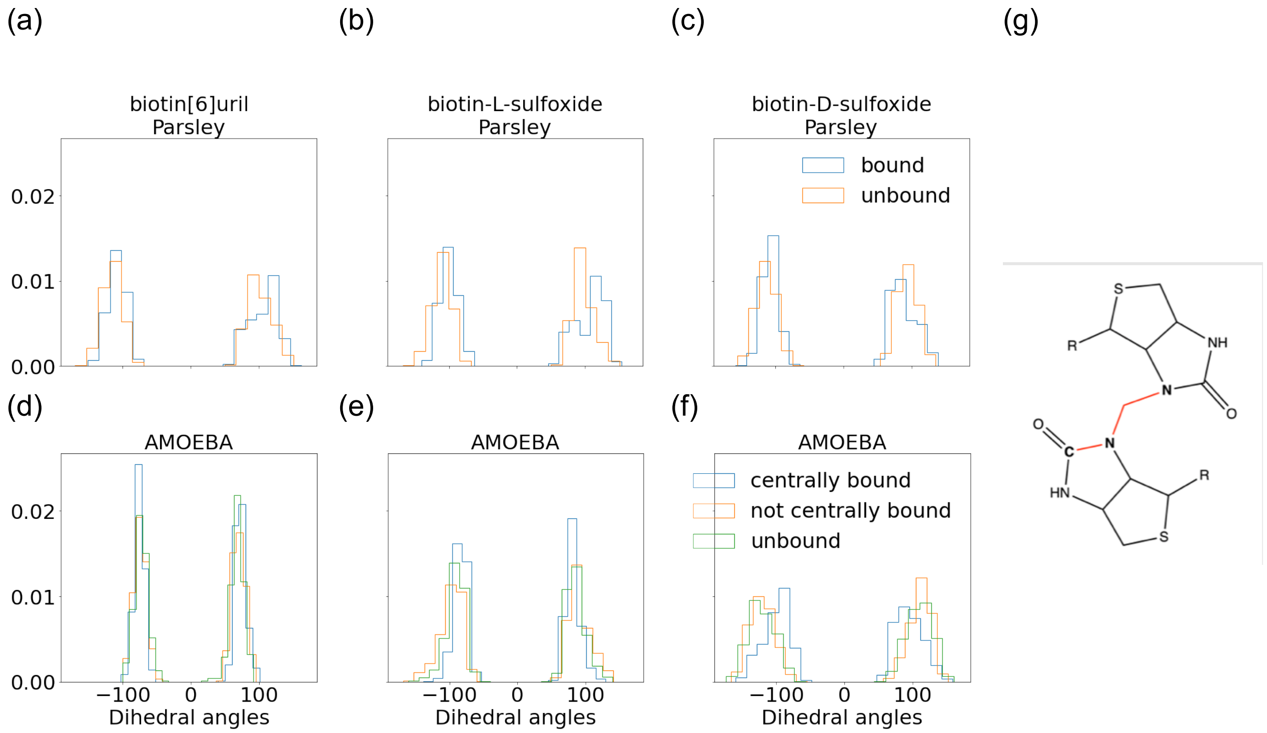

Figure S5. *C-N-C-N Dihedral distributions for three different biotin macrocycles in water with two different forcefields and different binding states. The C-N-C-N dihedral (highlighted in red) in (g) spans atoms from different sub-units and the second carbon atom is the carbon linking two biotin subunits.*

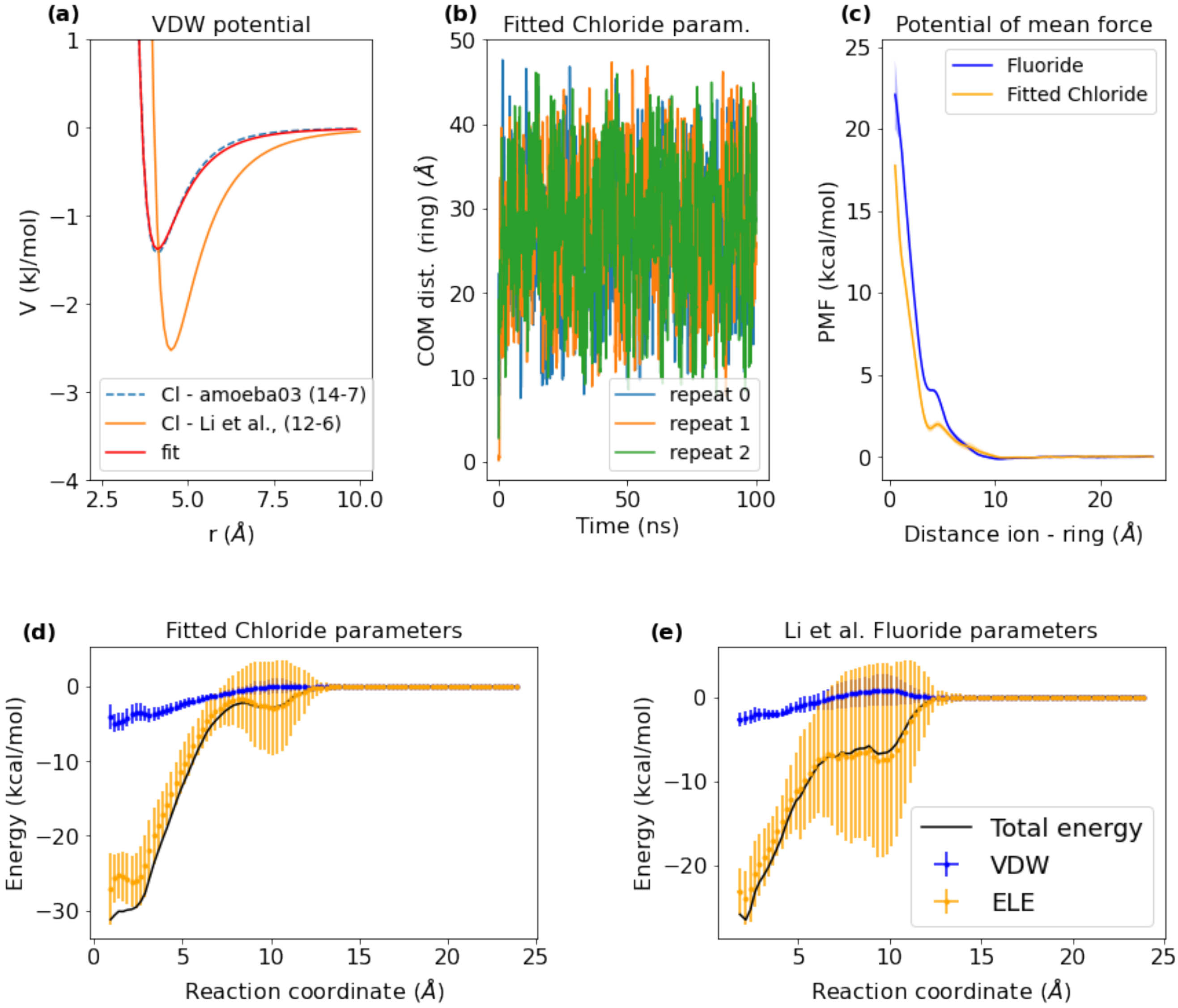

Figure S6. *Van der Waals potential for chloride. (a) Modifying the chloride parameters by Lennard-Jones fitting to resemble more closely the AMOEBA buffered 14-7 potential does not preserve the hydration free energy of chloride in water. The potential is depicted for a chloride-chloride pair. (b) The chloride with modified parameters is not stably bound in the cavities of the macrocycles. (c) The potential of mean force profile for Fluoride (Li et al. parameters) and for the modified chloride indicate that neither ion can stably bind to the cavity despite their smaller VDW radius. (d) Interaction energies between the biotin[6]uril host and the Li et al fluoride ion. (e) Interaction energies between the biotin[6]uril host and fluoride with the Li* et al. *parameters.*

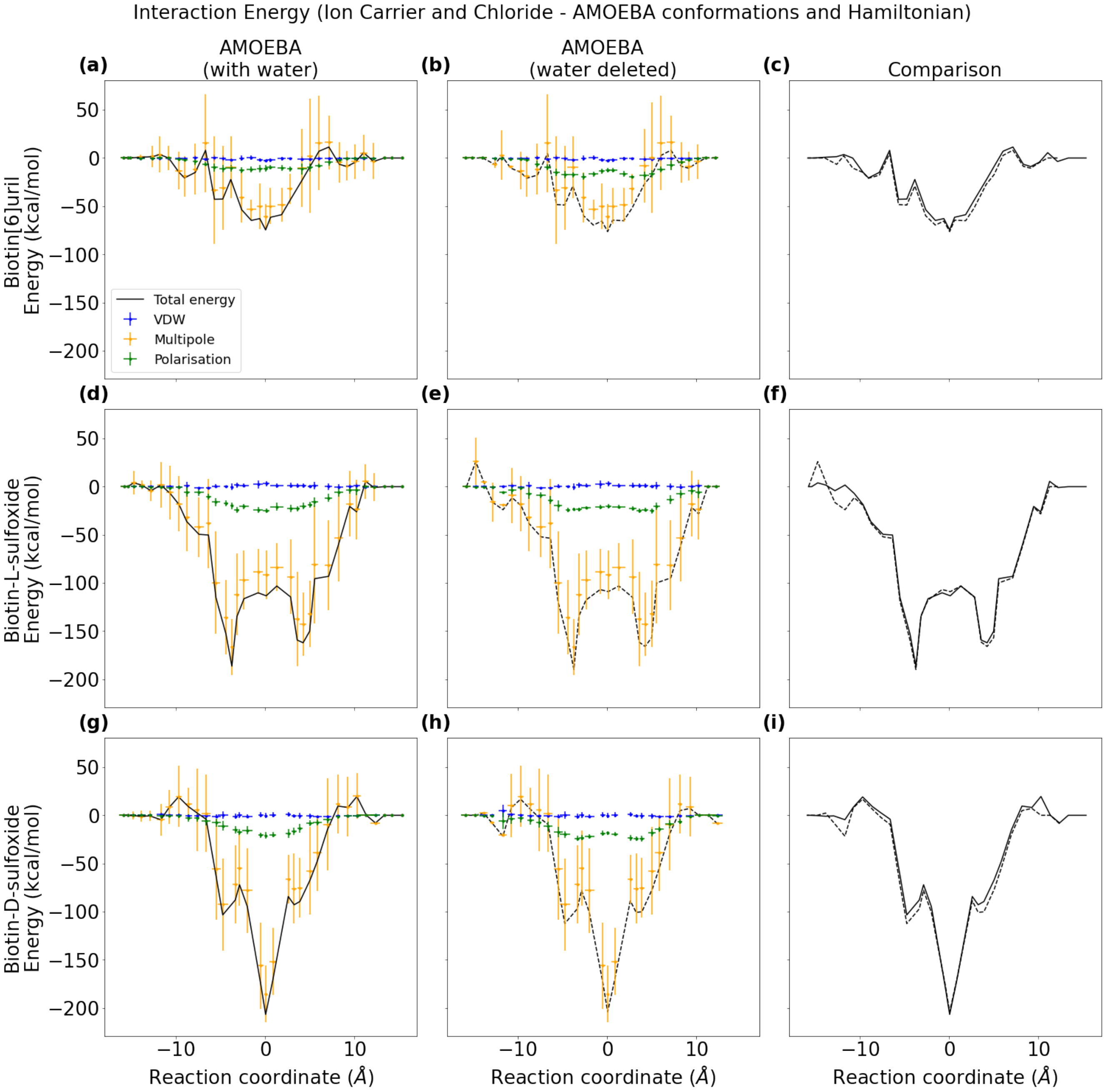

Figure S7. *Interaction energies along the reaction coordinate for three ion carriers and chloride in water evaluated from AMOEBA conformations with (a, d and g) and without water (b, e and h) with the AMOEBA force field. A direct comparison of the total energy is also shown (c, f and i). For each 13 ns long umbrella window, 13 conformations were selected to evaluate the interaction energy.*

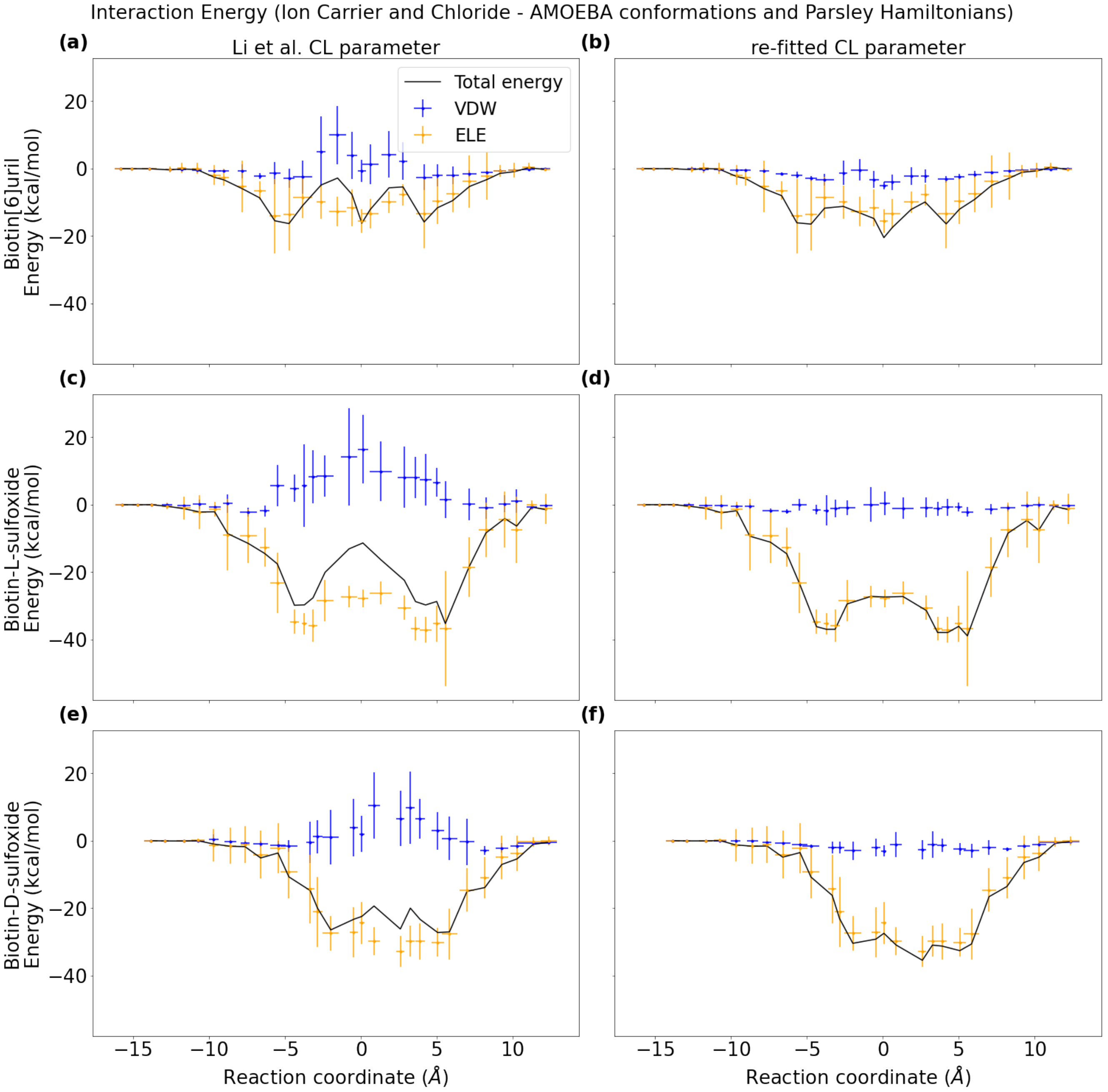

Figure S8. *Interaction energies along the reaction coordinate for three ion carriers and chloride in water evaluated from AMOEBA conformations with the Parsley force field for the host and two different chloride parameter sets. For each 13 ns long umbrella window, 13 conformations were selected to evaluate the interaction energy. The Li et al. parameter set and the modified/re-fitted chloride parameter set are compared in Figure S4.*

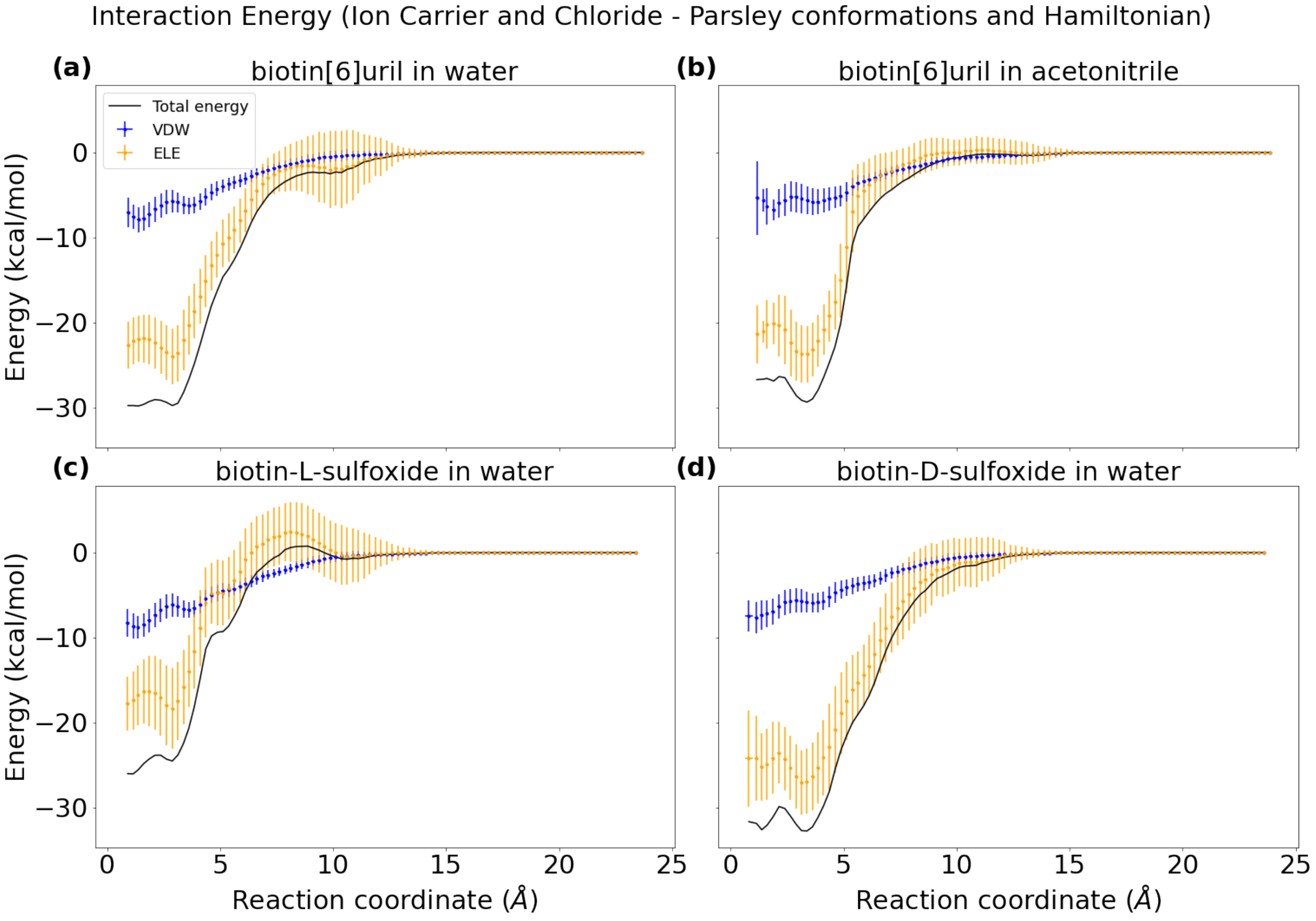

Figure S9. *Interaction energies along the reaction coordinate for three ion carriers and chloride evaluated from Parsley conformations with the Parsley force field for the host and the Li et al. chloride parameter set. For each 25ns long umbrella window, 1250 conformations were selected to evaluate the interaction energy.*

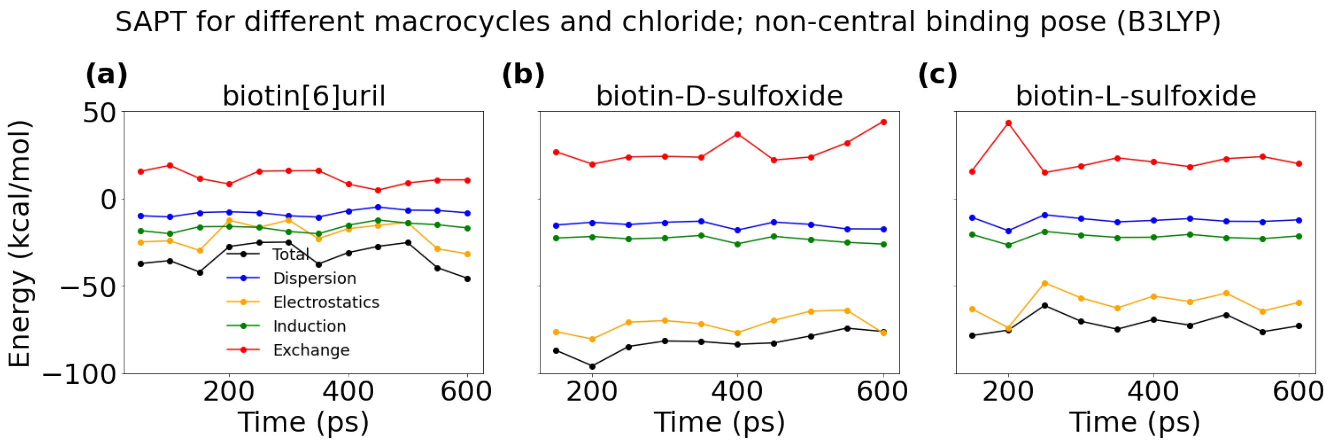

Figure S10. *Interaction energies between chloride and three hosts (biotin[6]uril, biotin-D-sulfoxide[6]uril and biotin-L-sulfoxide[6]uril). For ten AMOEBA frames and the non-central binding pose, we evaluated the interaction energy with symmetry-adapted perturbation theory (SAPT) on B3LYP level of theory with a jun-cc-pVDZ basis set.*

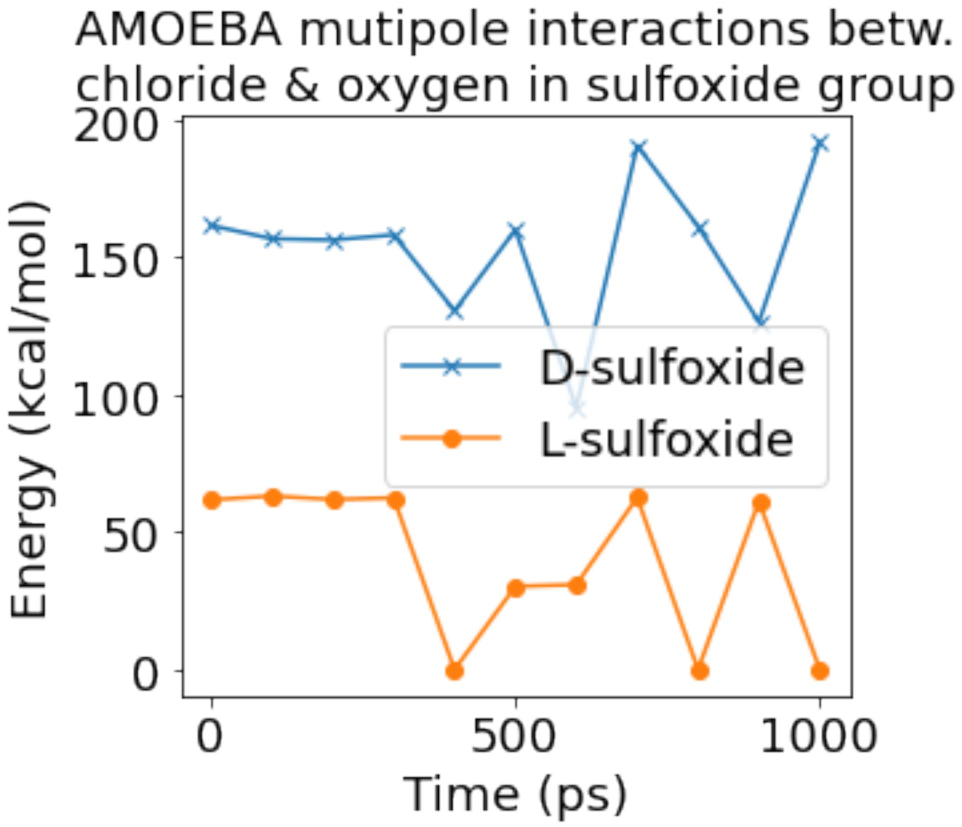

Figure S11. *Multipole interactions between chloride and the oxygens in the sulfoxide groups.*

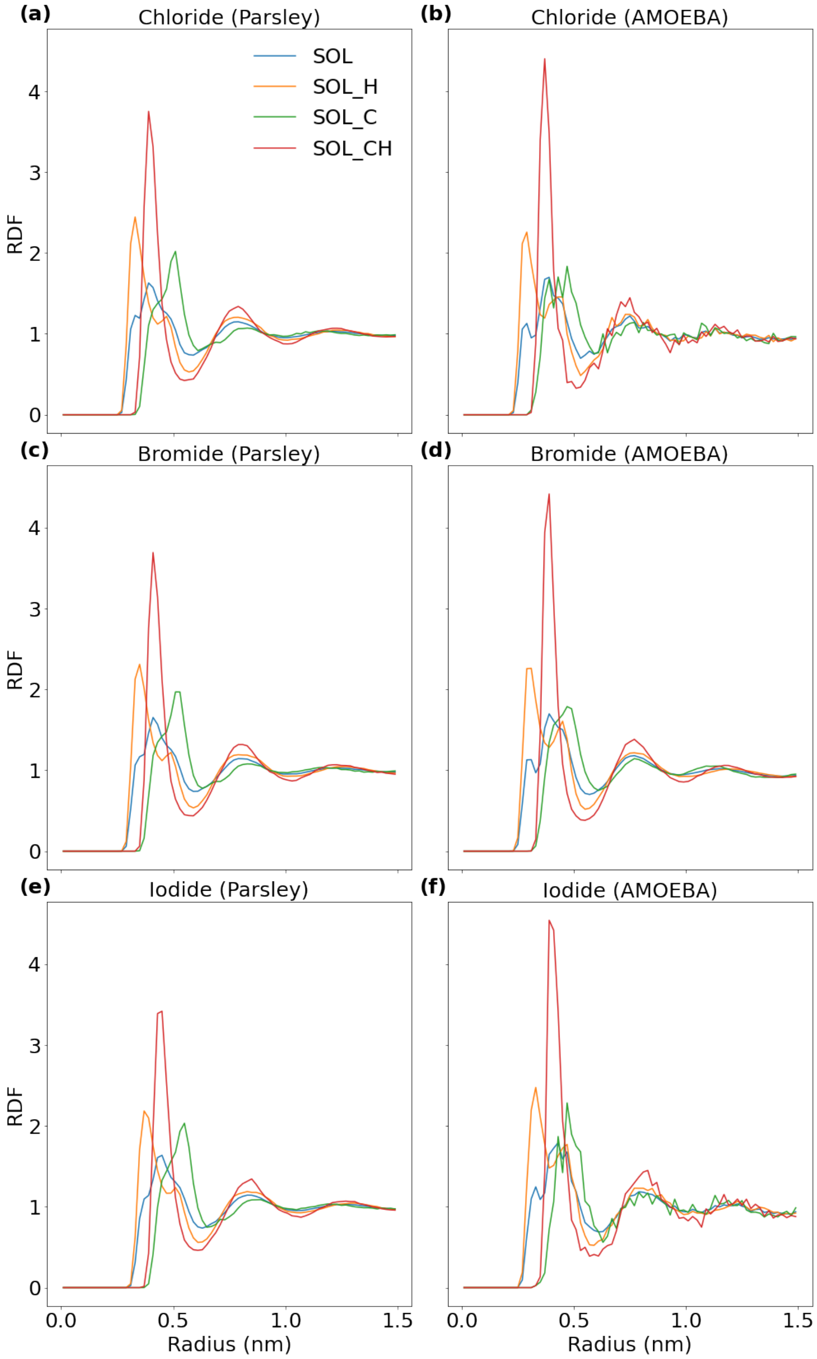

Figure S12. *Radial distribution functions (RDF) for halide anions and acetonitrile. SOL refers to the whole acetonitrile molecules, H refers to hydrogen, C to the carbon bound to nitrogen and CH to the carbon bound to three hydrogens. The RDFs are used to determine the cut-offs used in Table. S3 to analyze the hydration shell in umbrella windows.*

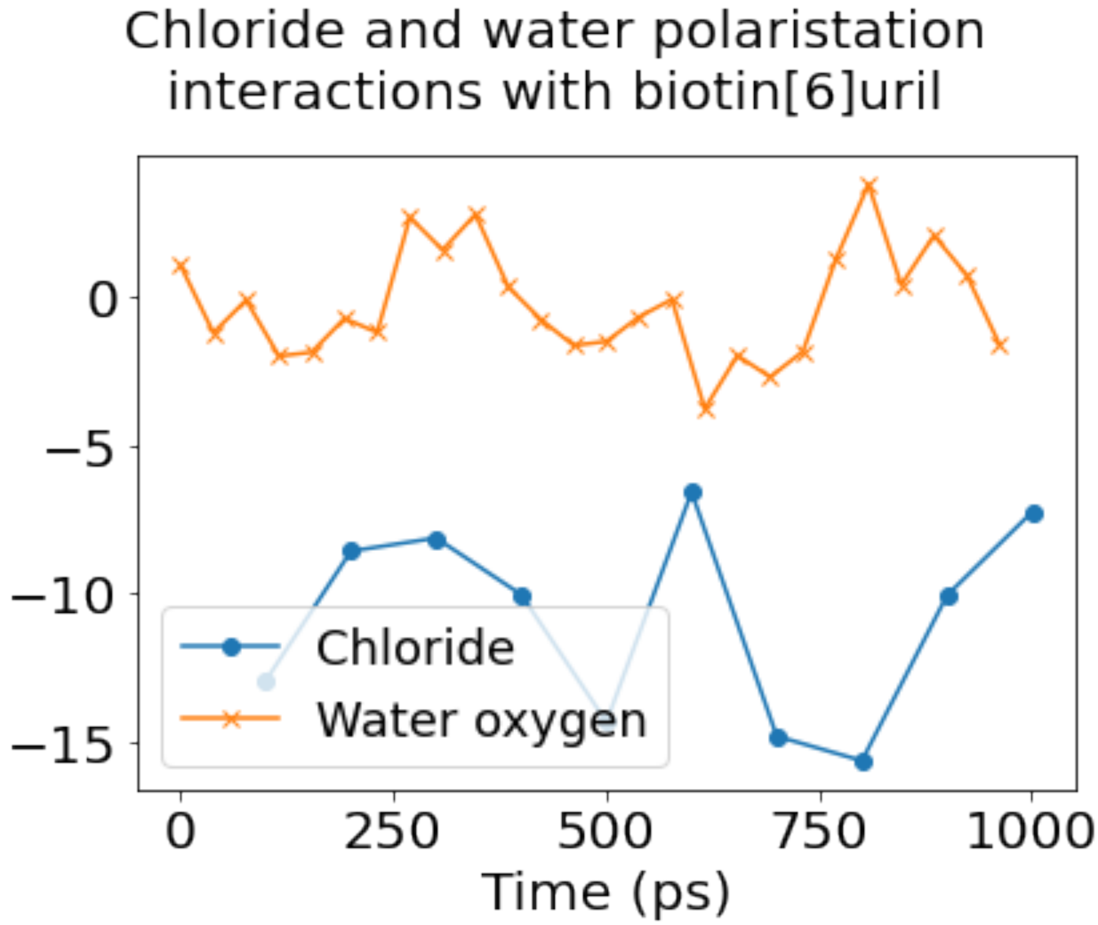

Figure S13. *Interaction energies between chloride and the biotin[6]uril macrocyle and a water molecule bound in the cavity and the macrocycle. The guests are both centrally bound in the cavity. As expected, the chloride has a stronger polarization interaction with the host than a water molecule.*

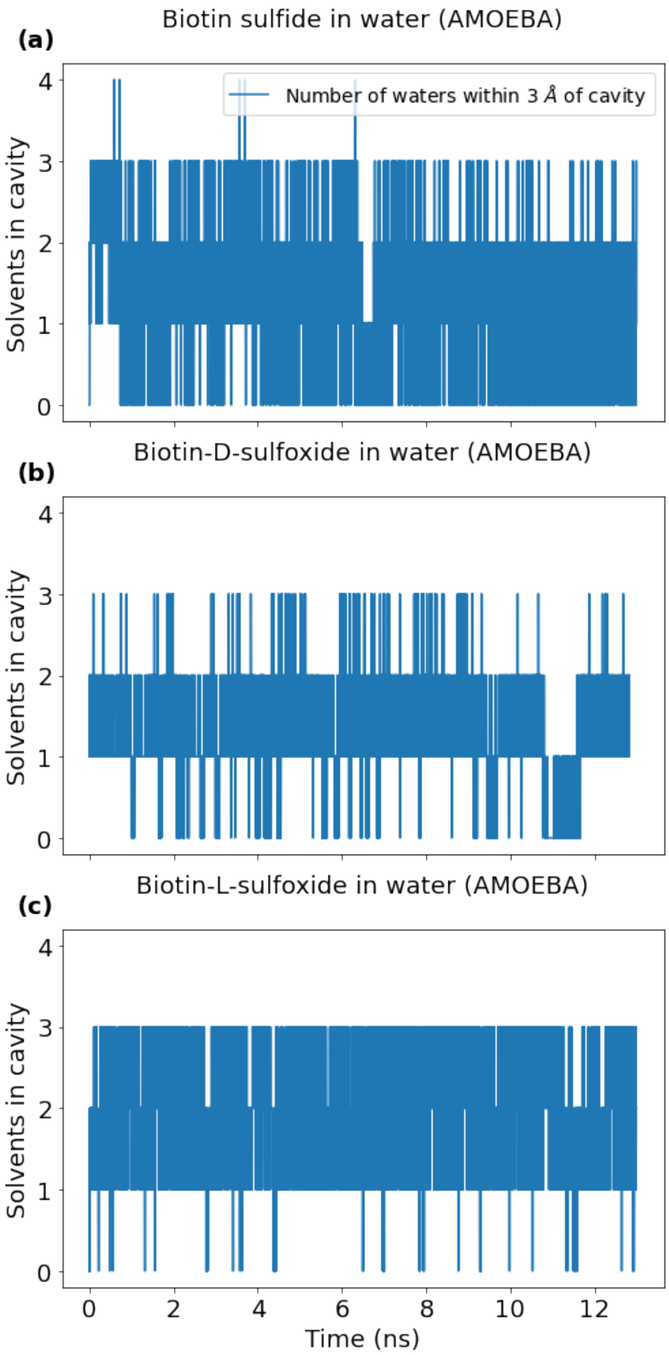

Figure S14. *Time series of number of solvent molecules within 3 Å of the cavity center for three different biotin macrocycles in water. A representative snap-shot of a hydrated and a de-wetted cavity are shown in Fig. 10 of the main manuscript.*

Table S1. Absolute Single-Ion Solvation Free Energies (kcal/mol) *in water and in Acetonitrile[19].*

Ion	Water	Acetonitrile
Chloride	−74.5	−62.4
Bromide	−68.3	−59.3
Iodide	−59.9	−53.0

Table S2 **Interaction energies in kcal/mol for chloride bound to different biotin macrocycles in water**. *For ten AMOEBA frames, we evaluated the interaction energy with symmetry-adapted perturbation theory (SAPT) on B3LYP level of theory with a jun-cc-pVDZ basis set.*

**Macrocycle**	**Interaction energy**	**Dispersion**	**Electrostatics**	**Induction**	**Exchange**
**Biotin[6]uril**	-56.6 ± 4.5	-17.0 ± 1.1	-41.6 ± 5.3	-23.3 ± 1.1	25.3 ± 3.9
**Biotin-D-sulfoxide**	-87.8 ± 4.9	-22.1 ± 1.3	-79.3 ± 5.8	-28.3 ± 1.0	42.0 ± 4.7
**Biotin-L-sulfoxide**	-87.8 ± 4.5	-19.6 ± 1.4	-76.5 ± 4.3	-26.0 ± 1.3	34.4 ± 5.8

Table S3. **AMOEBA interaction energies in kcal/mol for chloride bound to different biotin macrocycles in water.** *For ten AMOEBA frames, we performed energy decomposition analysis with the AMOEBA force field.*

Macrocycle	Interaction energy	VDW	Electrostatics	Polarisation
**Biotin[6]uril**	-75.1 ± 12.3	-3.3 ± 1.5	-60.9 ± 11.1	-10.8 ± 3.2
**Biotin-D-sulfoxide**	-110.9 ± 19.2	2.4 ± 2.0	-89.2 ± 19.0	-24.1 ± 2.2
**Biotin-L-sulfoxide**	-208.2 ± 24.1	0.2 ± 2.6	-189.4 ± 23.0	-19.0 ± 2.5

Table S4. Hydration shell analysis. *Cut-off distances for counting ion interactions with biotin, acetonitrile and water in. The cut-offs are determined by analyzing the radial distribution functions (RDF) of the respective ion with the respective solvent as depicted in Fig. S3.*

	Distances in Å	Chloride	Bromide	Iodide
**Biotin**	Cutoff distance for interactions	3.0	3.25	3.5
**Acetonitrile**	End of 1st solvation shell (C-H3)	5.5	5.9	6.2
	End of 1st solvation shell (hydrogen)	4.4	4.5	4.7
**Water**	End of 1st solvation shell (oxygen)	3.75	4.0	4.25
	End of 1st solvation shell (hydrogen)	2.75	3.0	3.25

Table S5. Comparison of gromacs and tinker simulation performance*.

	Box size	*Performance*
Gromacs 2020.3	18029 atoms	308 ns/day
Tinker9	9089 atoms	38.7 ns/day

*Using a single A40 GPU and 6 cores of an AMD 7402 2.8GHz node.

REFERENCES

1. J. Wang, R. M. Wolf, J. W. Caldwell, P. A. Kollman and D. A. Case, *J. Comp. Chem.*, 2004, **25**, 1157-1174.
2. J. Wagner, M. Thompson, D. Dotson, H. Jang, S. Boothroyd and J. Rodríguez-Guerra, *Journal*, 2021, DOI: https://doi.org/10.5281/ZENODO.5009058.
3. J. Wang, W. Wang, P. A. Kollman and D. A. Case, *J. Mol. Graph. Mod.*, 2006, **25**, 247-260.
4. W. L. Jorgensen, J. Chandresekhar, J. D. Madura, R. W. Impey and M. L. Klein, *J. Chem. Phys.*, 1983, **79**, 926-935.
5. E. J. Sorin and V. S. Pande, *Biophys. J.*, 2005, **88**, 2472-2493.
6. P. Li, L. F. Song and K. M. Merz, Jr., *J. Chem. Theory Comput.*, 2015, **11**, 1645-1657.
7. P. Ren, C. Wu and J. W. Ponder, *J. Chem. Theory Comput.*, 2011, **7**, 3143-3161.
8. P. Ren and J. W. Ponder, *J. Phys. Chem. B*, 2003, **107**, 5933-5947.
9. J. C. Wu, G. Chattree and P. Ren, *Theor. Chem. Acc.*, 2012, **131**, 1138.
10. M. L. Laury, Z. Wang, A. S. Gordon and J. W. Ponder, *J. Comput-Aided Mol. Des.*, 2018, **32**, 1087-1095.
11. A. J. Stone, *J. Chem. Theory Comput.*, 2005, **1**, 1128-1132.
12. P. Ren and J. W. Ponder, *J. Comp. Chem.*, 2002, **23**, 1497-1506.
13. J. A. Rackers, Z. Wang, C. Lu, M. L. Laury, L. Lagardère, M. J. Schnieders, J.-P. Piquemal, P. Ren and J. W. Ponder, *J. Chem. Theory Comput.*, 2018, **14**, 5273-5289.
14. Y. Zhang, Y. Jiang, Y. Qiu and H. Zhang, *J. Chem. Inf. Model.*, 2021, **61**, 4613-4629.
15. M. Smith, Z. Li, L. Landry, K. M. Merz and P. Li, *ChemRxiv.*, 2023.
16. H. D. Lim VT, Tresadern G et al. [version 1; peer review: 2 approved]. F1000Research 2020, 9(Chem Inf Sci):1390 *F1000Research 2020, 9(Chem Inf Sci)*, 2020, **9**, 1390.
17. J. W. Ponder, C. Wu, P. Ren, V. S. Pande, J. D. Chodera, M. J. Schnieders, I. Haque, D. L. Mobley, D. S. Lambrecht, R. A. DiStasio, M. Head-Gordon, G. N. I. Clark, M. E. Johnson and T. Head-Gordon, *J. Phys. Chem. B*, 2010, **114**, 2549-2564.
18. B. T. Thole, *Chem. Phys.*, 1981, **59**, 341-350.
19. C. P. Kelly, C. J. Cramer and D. G. Truhlar, *The Journal of Physical Chemistry B*, 2007, **111**, 408-422.
